# Supplementary material for: Choosing important health outcomes for comparative effectiveness research: 6th annual update to a systematic review of core outcome sets for research
Source: PLoS One. 2021 Jan 12;16(1):e0244878. doi: 10.1371/journal.pone.0244878 (PMC7802923; doi:10.1371/journal.pone.0244878)
Supplement: S1 Table — (DOCX) [file pone.0244878.s002.docx]

**S1 Table. Search strategy**

| **Search terms for MEDLINE** | | |
| --- | --- | --- |
| 1 | Health Services/ut [Utilization] | |
| 2 | registries/ | |
| 3 | systematic review.mp. | |
| 4 | structured review.ti. | |
| 5 | evidence based medicine.ab. | |
| 6 | exp Clinical Trials as Topic/ | |
| 7 | clinical trial$.ab. | |
| 8 | randomised controlled trial$.ti,ab. | |
| 9 | 1 or 2 or 3 or 4 or 5 or 6 or 7 or 8 or 9 | |
| 10 | workgroup$.mp. | |
| 11 | standard$ outcome$.mp. | |
| 12 | Practice Guideline/ | |
| 13 | clinical database.mp. | |
| 14 | patient important outcome$.mp. | |
| 15 | (standard$ adj3 reporting).mp. | |
| 16 | congresses.pt. | |
| 17 | Delphi Technique/ | |
| 18 | (recommend$ adj3 outcome$).mp. | |
| 19 | consensus development conference.pt. | |
| 20 | outcome$ reporting.mp. | |
| 21 | priorit$ symptom$.mp. | |
| 22 | (task force adj3 outcome$).mp. | |
| 23 | appropriate outcome$.mp. | |
| 24 | research design/ |  |
| 25 | endpoint determination/ | |
| 26 | consensus development conference/ | |
| 27 | patient participation/ | |
| 28 | consensus.mp. | |
| 29 | workshop.mp. | |
| 30 | Consensus Development Conferences, NIH as Topic/ | |
| 31 | focus groups/ | |
| 32 | 11 or 12 or 13 or 14 or 15 or 16 or 17 or 18 or 19 or 20 or 21 or 22 or 23 or 24 or 25 or 26 or 27 or 28 or 29 or 30 or 31 | |
| 33 | outcome$.mp. | |
| 34 | end point$.mp. | |
| 35 | (core adj3 set).mp. | |
| 36 | treatment emergent problem$.mp. | |
| 37 | exp outcome Assessment Health Care/ | |
| 38 | Treatment Outcome/ | |
| 39 | Quality of Life/ | |
| 40 | 34 or 35 or 36 or 37 or 38 or 39 | |
| 41 | clinical-study design.mp. | |
| 42 | patient$ perspective$.ti. | |
| 43 | outcome$.mp. and delphi.ti. | |
| 44 | (outcome$ and delphi).ab. | |
| 45 | (perspective$ adj3 outcome$).ti. | |
| 46 | core outcome$.ti,ab. | |
| 47 | core set$.ti,ab. | |
| 48 | clinical trial design$.ti. | |
| 49 | design$ clinical trial$.ti. | |
| 50 | (consensus and outcome$).ti. | |
| 51 | 42 or 43 or 44 or 45 or 46 or 47 or 48 or 49 or 50 | |
| 52 | 9 and 32 and 40 | |
| 53 | 51 or 52 | |
|  | limit to ed=20190101-20191231 | |
| **Search terms for SCOPUS** | | |
| ( ( ( ( INDEXTERMS ( *registries* ) )  OR  ( INDEXTERMS ( *clinical*  AND *trials*  AND *as*  AND *topic* ) )  OR  ( ABS ( *"evidence based medicine"* ) )  OR  ( ABS ( *"clinical trial*"* ) )  OR  ( INDEXTERMS ( *"Health Services Utilization"* ) )  OR  ( TITLE-ABS-KEY ( *"SYSTEMATIC REVIEW"* ) )  OR  ( TITLE ( *"structured review"* ) ) )  OR  ( *title*  OR  ABS ( *"randomised controlled trial*"* ) ) )  AND  ( ( ( TITLE-ABS-KEY ( *workgroup** ) )  OR  ( TITLE-ABS-KEY ( *standard**  AND *outcome** ) )  OR  ( INDEXTERMS ( *practice*  AND *guideline* ) )  OR  ( TITLE-ABS-KEY ( *"clinical database"* ) )  OR  ( TITLE-ABS-KEY ( *"patient important outcome*"* ) )  OR  ( TITLE-ABS-KEY ( *"standard* outcome*"* ) )  OR  ( INDEXTERMS ( *delphi*  AND *technique* ) ) )  OR  ( ( TITLE-ABS-KEY ( *recommend**  W/3  *outcome** ) )  OR  ( TITLE-ABS-KEY ( *standard**  W/3  *reporting** ) )  OR  ( TITLE-ABS-KEY ( *task*  AND *force*  W/3  *outcome** ) )  OR  ( TITLE-ABS-KEY ( *"appropriate outcome*"* ) )  OR  ( TITLE-ABS-KEY ( *"outcome* reporting"* ) )  OR  ( TITLE-ABS-KEY ( *"priorit* symptom*"* ) )  OR  ( INDEXTERMS ( *focus*  AND *group* ) )  ( INDEXTERMS ( *research*  AND *design* ) ) )  OR  ( ( INDEXTERMS ( *endpoint*  AND *determination* ) )  OR  ( INDEXTERMS ( *consensus*  AND *development*  AND *conference* ) )  OR  ( INDEXTERMS ( *patient*  AND *participation* ) )  OR  ( TITLE-ABS-KEY ( *consensus* ) )  OR  ( TITLE-ABS-KEY ( *workshop* ) ) ) )  AND  *74* )  OR  ( ( ( TITLE ( *"design* clinical trials"* ) )  OR  ( TITLE ( *consensus*  AND  *outcome** ) )  OR  ( TITLE-ABS-KEY ( *"clinical-study design"* ) )  OR  ( TITLE ( *"patient* perspective*"* ) )  OR  ( ABS ( *outcome**  AND  *delphi* ) )  OR  ( TITLE ( *outcome**  AND  *delphi* ) )  OR  ( TITLE ( *perspective**  W/3  *outcome** ) )  OR  ( ABS ( *"core outcome*"* )  OR  TITLE ( *"core outcome*"* ) ) )  OR  ( ( ABS ( *"core set*"* )  OR  TITLE ( *"core set*"* ) )  OR  ( TITLE ( *"clinical trial design*"* ) ) ) )  AND  ORIG-LOAD-DATE  >  *20190101*  AND  ORIG-LOAD-DATE  <  *20191231* | | |
