# Supplementary material for: Choosing important health outcomes for comparative effectiveness research: 6th annual update to a systematic review of core outcome sets for research
Source: PLoS One. 2021 Jan 12;16(1):e0244878. doi: 10.1371/journal.pone.0244878 (PMC7802923; doi:10.1371/journal.pone.0244878)
Supplement: S2 Table — (DOCX) [file pone.0244878.s003.docx]

**S2 Table. Table of reports included in updated review (n=81)**

| **Study** | **Disease category** | **Disease name** |
| --- | --- | --- |
| Audigé 2019 [1]**  Jacxsens 2017 [2] | Orthopaedics & trauma | Shoulder arthroplasty |
| Blackwood 2019 [3]**  Blackwood 2014 [4] | Lungs & airways | Mechanical ventilation |
| Bogdanet 2019 [5]** | Pregnancy & childbirth | Gestational diabetes mellitus |
| Chen 2019 [6]** | Orthopaedics & trauma | Whiplash-associated disorder |
| Crudgington 2019 [7]** | Neurology | Rolandic epilepsy |
| de Wolf-Linder 2019 [8]** | Other | N/A – palliative care |
| Doumouchtsis 2019 [9]** | Gynaecology | Female stress urinary incontinence |
| *Duarte-Garcia 2019 [10]^*  *Leung 2019 [11]^*  *Orbai 2019 [12]^*  *Eder 2013 [13]^* | *Rheumatology* | *Psoriatic Arthritis* |
| *Foeldvari 2019 [14]^* | *Rheumatology* | *JIA-associated uveitis* |
| Gaba 2016 [15]* | Cancer | Hepatic malignancy |
| Goncalves 2019 [16]**  Goncalves 2019 [17]  Goncalves 2018 [18] | Neurology | Dementia |
| *Grinich 2018 [19]^*  *Howells 2019 [20]^*  *Schmitt 2014 [21]^* | *Skin* | *Eczema* |
| Haller 2019 [22]** | Other | Perioperative medicine |
| Harman 2019 [23]**  Harman 2017 [24]  Gorst 2019 [25] | Endocrine & metabolic | Type 2 diabetes |
| Healy 2019 [26]** | Pregnancy & childbirth | Fetal growth restriction (FGR) |
| Hinkelbein 2019 [27]** | Lungs & airways; Anaesthesia & pain control | Airway management |
| Hodgson 2019 [28]** | Heart & circulation | Cardiac and respiratory failure in critically ill patients |
| *Horbach 2019 [29]^*  *Lokhorst 2019 [30]^* | *Heart & circulation; Skin* | *Peripheral vascular malformations* |
| Ingoe 2019 [31]** | Orthopaedics & trauma | Rib fracture/severe chest trauma |
| Joachim 2019 [32]**  Kapadia 2016 [33] | Neurology; Gastroenterology; Child health | Neurological impairment |
| *Ju 2019 [34]^* | *Kidney disease* | *Kidney transplant* |
| Krezel 2019 [35]**  Krezel 2015 [36]  Krezel 2015 [37] | Eyes & vision | Geographic atrophy (advanced form of Age-related macular degeneration (AMD)) |
| Kuizenga-Wessel 2017 [38]**  Kuizenga-Wessel 2015 [39]  Kuizenga-Wessel 2016 [40] | Gastroenterology | Functional constipation^a^ |
| Lam 2019 [41]** | Cancer | Prostate cancer (localised) |
| Mackenzie 2019 [42]** | Public health;  Endocrine & metabolic | Overweight/obesity |
| *Milman 2019 [43]^* | *Rheumatology* | *ANCA-associated vasculitis* |
| *Morgan 2019 [44]^* | *Rheumatology* | *Juvenile idiopathic arthritis* |
| *Owen 2019 [45]^* | *Rheumatology* | *Polymyalgia rheumatica* |
| *Perez-Chada 2019 [46]^* | *Skin* | *Psoriasis* |
| Perry 2019 [47]**  Perry 2018 [48] | Pregnancy & childbirth | Twin–twin transfusion syndrome (TTTS)^b^ |
| Pomponio 2019 [49]* | Wounds | Chronic wound infection |
| Ramiro 2019 [50]^ | Orthopaedics & trauma | Shoulder disorders |
| Regardt 2019 [51]**  Alexanderson 2014 [52]  Park 2017 [53]  Regardt 2015 [54]  Mecoli 2019 [55] | Rheumatology | Myositis |
| Rowe 2019 [56]** | Neurology; Eyes & vision | Stroke^c^ |
| Shorter 2019 [57]**  Shorter 2019 [58] | Tobacco, drugs & alcohol dependence | Alcohol problems |
| *Smith 2019 [59]^* | *Rheumatology* | *Hip and/or knee osteoarthritis* |
| Sun 2019 [60]** | Blood disorders | Haemophilia |
| *Thiboutot 2019 [61]^* | *Skin* | *Acne* |
| Tong 2018 [62]**  Sautenet 2018 [63]  Urquhart-Secord 2016 [64]  Evangelidis 2017 [65]  Tong 2017 [66]  Ju 2018 [67]  Ju 2019 [68]  Ju 2018 [69]  Viecelli 2018 [70]  Viecelli 2018 [71] | Kidney Disease | Chronic kidney disease |
| Townsend 2019 [72]** | Pregnancy & childbirth | Selective fetal growth restriction in monochorionic twins |
| Van Rijssen 2019 [73]** | Cancer | Pancreatic cancer |
| van Tol 2019 [74]**  van Tol 2018 [75] | Gastroenterology | Haemorrhoidal disease |
| Vincenzino 2019 [76]** | Orthopaedics & trauma | Tendinopathy |
| *Wallace 2019 [77]^* | *Neurology* | *Aphasia* |
| Webbe 2019 [78]**  Webbe 2018 [79]  Webbe 2019 [80] | Neonatal care | N/A |
| Xue 2019 [81]** | Orthopaedics & trauma | Osteonecrosis of the femoral head (ONFH) |

*^ Linked to COS included in previous review (n=19)*

** Considered outcomes while addressing wider clinical trial design issues (n=2)*

*** Specifically considered outcome selection and measurement (n=31)*

*^a^ Reports the development of two COS for functional constipation: (i) infants 0-1 years; (ii) children 1-18 years.*

*^b^ Reports the development of three COS for twin–twin transfusion syndrome (TTTS): (i) fetal outcomes; (ii) neonatal outcomes; (iii) maternal outcomes.*

*^c^ Reports the development of two COS for stroke: (i) vision screening; (ii) full vision assessment.*

**References**

1. Audigé L, Schwyzer HK; Shoulder Arthroplasty Core Event Set (SA CES) Consensus Panel, Durchholz H. Core set of unfavorable events of shoulder arthroplasty: an international Delphi consensus process. J Shoulder Elbow Surg. 2019;28(11):2061-2071. doi:10.1016/j.jse.2019.07.021
2. Jacxsens M, Walz T, Durchholz H, et al. Towards standardised definitions of shoulder arthroplasty complications: a systematic review of terms and definitions. Arch Orthop Trauma Surg. 2017;137(3):347-355. doi:10.1007/s00402-017-2635-9
3. Blackwood B, Ringrow S, Clarke M, et al. A Core Outcome Set for Critical Care Ventilation Trials. Crit Care Med. 2019;47(10):1324-1331. doi:10.1097/CCM.0000000000003904
4. Blackwood B, Clarke M, McAuley D, et al. How Outcomes Are Defined in Clinical Trials of Mechanically Ventilated Adults and Children. American Journal of Respiratory and Critical Care Medicine. 2014;189(8):886-893. doi: 10.1164/rccm.201309-1645PP
5. Bogdanet D, Reddin C, Macken E, et al. Follow-up at 1 year and beyond of women with gestational diabetes treated with insulin and/or oral glucose-lowering agents: a core outcome set using a Delphi survey. Diabetologia. 2019;62(11):2007-2016. doi:10.1007/s00125-019-4935-9
6. Chen K, Andersen T, Carroll L, et al. Recommendations For Core Outcome Domain Set For Whiplash-Associated Disorders (CATWAD). Clin J Pain. 2019;35(9):727-736. doi:10.1097/AJP.0000000000000735
7. Crudgington H, Rogers M, Bray L, et al. Core Health Outcomes in Childhood Epilepsy (CHOICE): Development of a core outcome set using systematic review methods and a Delphi survey consensus. Epilepsia. 2019;60(5):857-871. doi:10.1111/epi.14735
8. de Wolf-Linder S, Dawkins M, Wicks F, et al. Which outcome domains are important in palliative care and when? An international expert consensus workshop, using the nominal group technique. Palliat Med. 2019;33(8):1058-1068. doi:10.1177/0269216319854154
9. Doumouchtsis SK, Pookarnjanamorakot P, Durnea C, et al. A systematic review on outcome reporting in randomised controlled trials on surgical interventions for female stress urinary incontinence: a call to develop a core outcome set. BJOG. 2019;126:1417-1422. doi:10.1111/1471-0528.15891
10. Duarte-García A, Leung YY, Coates LC, et al. Endorsement of the 66/68 joint count for the measurement of musculoskeletal disease activity: OmeRACT 2018 Psoriatic Arthritis Workshop Report. Journal of Rheumatology. 2019;46(8):996-1005. doi:10.3899/jrheum.181089
11. Leung YY, Orbai AM, Ogdie A, et al. The GRAPPA-OMERACT Psoriatic Arthritis Working Group at the 2018 Annual Meeting: Report and Plan for Completing the Core Outcome Measurement Set. Journal of Rheumatology. 2019;95:33-37. doi:10.3899/jrheum.190122.
12. Orbai AM, Holland R, Leung YY, et al. PsAID12 provisionally endorsed at OMERACT 2018 as core outcome measure to assess psoriatic arthritis-specific health-related quality of life in clinical trials. Journal of Rheumatology. 2019;46(8):990-995. doi:10.3899/jrheum.181077
13. Eder L, Gladman DD. Outcome Measures in Psoriatic Disease. Current Dermatology Reports. 2013;2(3):164-171. doi:10.1007/s13671-013-0050-1
14. Foeldvari IJ, Klotsche J, Simonini G, et al. Proposal for a definition for response to treatment, inactive disease and damage for JIA associated uveitis based on the validation of a uveitis related JIA outcome measures from the Multinational Interdisciplinary Working Group for Uveitis in Childhood (MIWGUC). Pediatric rheumatology online journal. 2019;17(1):66. doi:10.1186/s12969-019-0345-2
15. Gaba RC, Lewandowski RJ, Hickey R, et al. Transcatheter Therapy for Hepatic Malignancy: Standardization of Terminology and Reporting Criteria. J Vasc Interv Radiol. 2016;27(4):457-473. doi:10.1016/j.jvir.2015.12.752
16. Gonçalves AC, Samuel D, Ramsay M, Demain S, Marques A. A Core Outcome Set to Evaluate Physical Activity Interventions for People Living With Dementia. Gerontologist. 2020;60(4):682-692. doi:10.1093/geront/gnz100
17. Gonçalves AC, Marques A, Samuel D, Demain S. Outcomes of physical activity for people living with dementia: qualitative study to inform a Core Outcome Set. Physiotherapy. 2019;S0031-9406(19)30072-0. doi:10.1016/j.physio.2019.05.003
18. Gonçalves AC, Cruz J, Marques A, Demain S, Samuel D. Evaluating physical activity in dementia: a systematic review of outcomes to inform the development of a core outcome set. Age Ageing. 2018;47(1):34-41. doi:10.1093/ageing/afx135
19. Grinich EE, Schmitt J, Küster D, et al. Standardized reporting of the Eczema Area and Severity Index (EASI) and the Patient-Oriented Eczema Measure (POEM): a recommendation by the Harmonising Outcome Measures for Eczema (HOME) Initiative. Br J Dermatol. 2018;179(2):540-541. doi:10.1111/bjd.16732
20. Schmitt J, Spuls PI, Thomas KS, et al. The Harmonising Outcome Measures for Eczema (HOME) statement to assess clinical signs of atopic eczema in trials. J Allergy Clin Immunol. 2014;134(4):800-807. doi:10.1016/j.jaci.2014.07.043
21. Howells L, Thomas KS, Sears AV, et al. Defining and measuring 'eczema control': an international qualitative study to explore the views of those living with and treating atopic eczema. J Eur Acad Dermatol Venereol. 2019;33(6):1124-1132. doi:10.1111/jdv.15475
22. Haller G, Bampoe S, Cook T, et al. Systematic review and consensus definitions for the Standardised Endpoints in Perioperative Medicine initiative: clinical indicators. Br J Anaesth. 2019;123(2):228-237. doi:10.1016/j.bja.2019.04.041
23. Harman NL, Wilding JPH, Curry D, et al. Selecting Core Outcomes for Randomised Effectiveness trials In Type 2 diabetes (SCORE-IT): a patient and healthcare professional consensus on a core outcome set for type 2 diabetes. BMJ Open Diab Res Care 2019;7:e000700. doi:10.1136/bmjdrc-2019-000700.
24. Harman NL, James R, Wilding J, et al. SCORE-IT (selecting core outcomes for randomised effectiveness trials in type 2 diabetes): a systematic review of registered trials. Trials 2017;18:597. doi:10.1186/s13063-017-2317-5
25. Gorst SL, Young B, Williamson PR, et al. Incorporating patients' perspectives into the initial stages of core outcome set development: a rapid review of qualitative studies of type 2 diabetes. BMJ Open Diabetes Res Care 2019;7:e000615. doi:10.1136/bmjdrc-2018-000615
26. Healy P, Gordijn SJ, Ganzevoort W, et al. A Core Outcome Set for the prevention and treatment of fetal GROwth restriction: deVeloping Endpoints: the COSGROVE study. Am J Obstet Gynecol. 2019;221(4):339.e1-339.e10. doi:10.1016/j.ajog.2019.05.039
27. Hinkelbein J, Iovino I, De Robertis E, Kranke P. Outcomes in video laryngoscopy studies from 2007 to 2017: systematic review and analysis of primary and secondary endpoints for a core set of outcomes in video laryngoscopy research. BMC Anesthesiol. 2019;19(1):47. doi:10.1186/s12871-019-0716-8
28. Hodgson CL, Burrell AJC, Engeler DM, et al. Core Outcome Measures for Research in Critically Ill Patients Receiving Extracorporeal Membrane Oxygenation for Acute Respiratory or Cardiac Failure: An International, Multidisciplinary, Modified Delphi Consensus Study. Crit Care Med. 2019;47(11):1557-1563. doi:10.1097/CCM.0000000000003954
29. Horbach SER, Rongen APM, Elbers RG, et al. Outcome measurement instruments for peripheral vascular malformations and an assessment of the measurement properties: a systematic review. Qual Life Res. 2020;29:1–17. doi:10.1007/s11136-019-02301-x
30. Lokhorst M, Horbach S, Waner M, et al. Responsiveness of quality‐of‐life measures in patients with peripheral vascular malformations: the OVAMA project. Br J Dermatol. 2020;182:1395-1403. doi:[10.1111/bjd.18619](https://doi.org/10.1111/bjd.18619)
31. Ingoe HMA, Eardley W, Rangan A, Hewitt C, McDaid C. An international multi-stakeholder delphi consensus exercise to develop a core outcomes set (COS) for surgical fixation of rib fractures. Injury. 2020;51(2):224-229. doi:10.1016/j.injury.2019.10.031
32. Joachim KC, Farid-Kapadia M, Butcher NJ, et al. Core outcome set for children with neurological impairment and tube feeding. Dev Med Child Neurol. 2020;62(2):201-206. doi:10.1111/dmcn.14326
33. Kapadia MZ, Joachim KC, Balasingham C, et al. A core outcome set for children with feeding tubes and neurologic impairment: a systematic review. Pediatrics. 2016;138:e20153967. doi:10.1542/peds.2015-3967
34. Ju A, Josephson MA, Butt Z, et al. Establishing a Core Outcome Measure for Life Participation: A Standardized Outcomes in Nephrology-kidney Transplantation Consensus Workshop Report. Transplantation. 2019;103(6):1199-1205. doi:10.1097/TP.0000000000002476
35. Krezel AK, Hogg R, Lohfeld L, Chakravarthy U, Azuara-Blanco A. Core outcomes for geographic atrophy trials. Br J Ophthalmol. 2019;bjophthalmol-2019-314949. doi:10.1136/bjophthalmol-2019-314949
36. Krezel AK, Hogg RE, Azuara-Blanco A. Patient-reported outcomes in randomised controlled trials on age-related macular degeneration. British Journal of Ophthalmology. 2015;99:1560-1564. doi:10.1136/bjophthalmol-2014-306544
37. Krezel AK, Hogg RE, Krezel S, Fallis R, Azuara-Blanco A. Design characteristic of randomised controlled trials for geographic atrophy in age-related macular degeneration: selection of outcomes and sample size calculation. Eye (Lond). 2015;29(11):1458-1463. doi:10.1038/eye.2015.132
38. Kuizenga-Wessel S, Steutel NF, Benninga MA, et al. Development of a core outcome set for clinical trials in childhood constipation: a study using a Delphi technique. BMJ Paediatrics Open. 2017;1:e000017. doi:10.1136/bmjpo-2017-000017
39. Kuizenga-Wessel S, Benninga MA, Tabbers MM. Reporting outcome measures of functional constipation in children from 0 to 4 years of age. J Pediatr Gastroenterol Nutr. 2015;60(4):446-456. doi:10.1097/MPG.0000000000000631
40. Kuizenga-Wessel S, Heckert SL, Tros W, van Etten-Jamaludin FS, Benninga MA, Tabbers MM. Reporting on Outcome Measures of Functional Constipation in Children-A Systematic Review. J Pediatr Gastroenterol Nutr. 2016;62(6):840-846. doi:10.1097/MPG.0000000000001110
41. Lam TBL, MacLennan S, Willemse PM, et al. EAU-EANM-ESTRO-ESUR-SIOG Prostate Cancer Guideline Panel Consensus Statements for Deferred Treatment with Curative Intent for Localised Prostate Cancer from an International Collaborative Study (DETECTIVE Study). Eur Urol. 2019;76(6):790-813. doi:10.1016/j.eururo.2019.09.020
42. Mackenzie RM, Ells LJ, Simpson SA, Logue J. Coreoutcome set for behavioural weight management interventions for adults with overweight and obesity: Standardised reporting of lifestyle weight management interventions to aid evaluation (STAR-LITE). Obes Rev. 2020;21(2):e12961. doi:10.1111/obr.12961
43. Milman N, McConville E, Robson JC, et al. Updating OMERACT Core Set of Domains for ANCA-associated Vasculitis: Patient Perspective Using the International Classification of Function, Disability, and Health. J Rheumatol. 2019;46(10):1415-1420. doi:10.3899/jrheum.181073
44. Morgan EM, Munro JE, Horonjeff J, et al. Establishing an Updated Core Domain Set for Studies in Juvenile Idiopathic Arthritis: A Report from the OMERACT 2018 JIA Workshop. J Rheumatol. 2019;46(8):1006-1013. doi:10.3899/jrheum.181088
45. Owen CE, Yates M, Twohig H, et al. Toward a Core Outcome Measurement Set for Polymyalgia Rheumatica: Report from the OMERACT 2018 Special Interest Group. J Rheumatol. 2019;46(10):1360-1364. doi:10.3899/jrheum.181050
46. Perez-Chada LM, Gottlieb AB, Cohen J, et al. Measuring psoriatic arthritis symptoms: A core domain in psoriasis clinical trials. J Am Acad Dermatol. 2020;82(1):54-61. doi:10.1016/j.jaad.2019.05.075
47. Perry H, Duffy JMN, Reed K, et al. Core outcome set for research studies evaluating treatments for twin-twin transfusion syndrome. Ultrasound Obstet Gynecol. 2019;54(2):255-261. doi:10.1002/uog.20183
48. Perry H, Duffy JMN, Umadia O, Khalil A; International Collaboration to Harmonise Outcomes for Twin-Twin Transfusion Syndrome (CHOOSE). Outcome reporting across randomized trials and observational studies evaluating treatments for twin-twin transfusion syndrome: systematic review. Ultrasound Obstet Gynecol. 2018;52(5):577-585. doi:10.1002/uog.19068
49. Pomponio G, Tedesco S, Peghetti A, et al. Improving the quality of clinical research on chronic wound infection treatment: Expert-based recommendations. Journal of Wound Care. 2019;28:S26-S31. doi.org/10.12968/jowc.2019.28.Sup1.S26
50. Ramiro S, Page MJ, Whittle SL, et al. The OMERACT core domain set for clinical trials of shoulder disorders. Journal of Rheumatology. 2019;46(8): 969-975. doi:10.3899/jrheum.181070
51. Regardt M, Mecoli CA, Park JK, et al. OMERACT 2018 Modified Patient-reported Outcome Domain Core Set in the Life Impact Area for Adult Idiopathic Inflammatory Myopathies. J Rheumatol. 2019;46(10):1351-1354. doi:10.3899/jrheum.181065
52. Alexanderson H, Del Grande M, Bingham CO 3rd, et al. Patient-reported outcomes and adult patients' disease experience in the idiopathic inflammatory myopathies. report from the OMERACT 11 Myositis Special Interest Group. J Rheumatol. 2014;41(3):581-592. doi:10.3899/jrheum.131247
53. Park JK, Mecoli CA, Alexanderson H, et al. Advancing the Development of Patient-reported Outcomes for Adult Myositis at OMERACT 2016: An International Delphi Study. J Rheumatol. 2018 Jul;45(7):1071. doi:10.3899/jrheum.161252.C2.
54. Regardt M, Basharat P, Christopher-Stine L, et al. Patients' Experience of Myositis and Further Validation of a Myositis-specific Patient Reported Outcome Measure - Establishing Core Domains and Expanding Patient Input on Clinical Assessment in Myositis. Report from OMERACT 12. J Rheumatol. 2015;42(12):2492-2495. doi:10.3899/jrheum.141243
55. Mecoli CA, Park JK, Alexanderson H, et al. Perceptions of Patients, Caregivers, and Healthcare Providers of Idiopathic Inflammatory Myopathies: An International OMERACT Study. J Rheumatol. 2019;46(1):106-111. doi:10.3899/jrheum.180353
56. Rowe FJ, Hepworth LR, Kirkham JJ. Development of core outcome sets for vision screening and assessment in stroke: a Delphi and consensus study. BMJ Open. 2019;9(9):e029578. doi:10.1136/bmjopen-2019-029578
57. Shorter GW, Heather N, Bray JW, et al. Prioritization of outcomes in efficacy and effectiveness of alcohol brief intervention trials: International multi-stakeholder e-delphi consensus study to inform a core outcome set. Journal of Studies on Alcohol and Drugs. 2019;80(3):299-309. doi:10.15288/jsad.2019.80.299
58. Shorter GW, Bray JW, Giles EL, et al. The variability of outcomes used in efficacy and effectiveness trials of alcohol brief interventions: A systematic review. Journal of Studies on Alcohol and Drugs. 2019;80(3): 286-298. doi:10.15288/jsad.2019.80.286
59. Smith TO, Hawker GA, Hunter DJ, et al. The OMERACT-OARSI Core Domain Set for Measurement in Clinical Trials of Hip and/or Knee Osteoarthritis. J Rheumatol. 2019;46(8):981-989. doi:10.3899/jrheum.181194
60. Sun HL, Breakey VR, Straatman L, Wu JK, Jackson S. Outcomes indicators and processes in transitional care in adolescents with haemophilia: A Delphi survey of Canadian haemophilia care providers. Haemophilia. 2019;25(2):296-305. doi:10.1111/hae.13699
61. Thiboutot DM, Layton AM, Chren MM, Eady EA, Tan J. Assessing effectiveness in acne clinical trials: steps towards a core outcome measure set. Br J Dermatol. 2019;181(4):700-706. doi:10.1111/bjd.18011
62. Tong A, Manns B, Wang AYM, et al. Implementing core outcomes in kidney disease: report of the Standardized Outcomes in Nephrology (SONG) implementation workshop. Kidney Int. 2018;94(6):1053-1068. doi:10.1016/j.kint.2018.08.018
63. Sautenet B, Tong A, Williams G, et al. Scope and Consistency of Outcomes Reported in Randomized Trials Conducted in Adults Receiving Hemodialysis: A Systematic Review. Am J Kidney Dis. 2018;72(1):62-74. doi:10.1053/j.ajkd.2017.11.010
64. Urquhart-Secord R, Craig JC, Hemmelgarn B, et al. Patient and Caregiver Priorities for Outcomes in Hemodialysis: An International Nominal Group Technique Study. Am J Kidney Dis. 2016;68(3):444-454. doi:10.1053/j.ajkd.2016.02.037
65. Evangelidis N, Tong A, Manns B, et al. Developing a set of core outcomes for trials in hemodialysis: an international Delphi survey. Am J Kidney Dis. 2017;70:464–475. doi:10.1053/j.ajkd.2016.11.029
66. Tong A, Manns B, Hemmelgarn B, et al. Establishing Core Outcome Domains in Hemodialysis: Report of the Standardized Outcomes in Nephrology-Hemodialysis (SONG-HD) Consensus Workshop. Am J Kidney Dis. 2017;69(1):97-107. doi:10.1053/j.ajkd.2016.05.022
67. Ju A, Unruh M, Davison S, et al. Establishing a Core Outcome Measure for Fatigue in Patients on Hemodialysis: A Standardized Outcomes in Nephrology-Hemodialysis (SONG-HD) Consensus Workshop Report. Am J Kidney Dis. 2018;72(1):104-112. doi:10.1053/j.ajkd.2017.12.018
68. Ju A, Unruh M, Davison SN, et al. Identifying dimensions of fatigue in haemodialysis important to patients, caregivers and health professionals: An international survey. Nephrology (Carlton). 2020;25(3):239-247. doi:10.1111/nep.13638
69. Ju A, Unruh ML, Davison SN, et al. Patient-Reported Outcome Measures for Fatigue in Patients on Hemodialysis: A Systematic Review. Am J Kidney Dis. 2018;71(3):327-343. doi:10.1053/j.ajkd.2017.08.019
70. Viecelli AK, O'Lone E, Sautenet B, et al. Vascular Access Outcomes Reported in Maintenance Hemodialysis Trials: A Systematic Review. Am J Kidney Dis. 2018;71(3):382-391. doi:10.1053/j.ajkd.2017.09.018
71. Viecelli AK, Tong A, O'Lone E, et al. Report of the Standardized Outcomes in Nephrology-Hemodialysis (SONG-HD) Consensus Workshop on Establishing a Core Outcome Measure for Hemodialysis Vascular Access. Am J Kidney Dis. 2018;71(5):690-700. doi:10.1053/j.ajkd.2017.12.003
72. Townsend R, Duffy JMN, Sileo F, et al. A core outcome set for studies investigating the management of selective fetal growth. Ultrasound Obstet Gynecol. 2020;55(5):652-660. doi: 10.1002/uog.20388
73. Van Rijssen LB, Gerritsen A, Henselmans I, et al. Core Set of Patient-reported Outcomes in Pancreatic Cancer (COPRAC): An International Delphi Study among Patients and Health Care Providers. Annals of Surgery. 2019;270(1):158-164. doi: 10.1097/SLA.0000000000002633
74. van Tol RR, Kimman ML, Melenhorst J, et al. European Society of Coloproctology Core Outcome Set for haemorrhoidal disease: an international Delphi study among healthcare professionals. Colorectal Dis. 2019;21(5):570-580. doi:10.1111/codi.14553
75. van Tol RR, van Zwietering E, Kleijnen J et al. Towards a core outcome set for hemorrhoidal disease – a systematic review of outcomes reported in literature. Int J Colorectal Dis. 2018;33:849-856. doi: 10.1007/s00384-018-3046-2
76. Vicenzino B, de Vos RJ, Alfredson H, et al. ICON 2019-International Scientific Tendinopathy Symposium Consensus: There are nine core health-related domains for tendinopathy (CORE DOMAINS): Delphi study of healthcare professionals and patients. Br J Sports Med. 2020;54(8):444-451. doi:10.1136/bjsports-2019-100894
77. Wallace SJ, Worrall L, Rose T, et al. A core outcome set for aphasia treatment research: The ROMA consensus statement. Int J Stroke. 2019;14(2):180-185. doi:10.1177/1747493018806200
78. Webbe JWH, Duffy JMN, Afonso E, et al. Core outcomes in neonatology: development of a core outcome set for neonatal research. Arch Dis Child Fetal Neonatal Ed. 2020;105(4):425-431. doi:10.1136/archdischild-2019-317501
79. Webbe J, Brunton G, Ali S, et al. Parent, patient and clinician perceptions of outcomes during and following neonatal care: a systematic review of qualitative research. BMJ Paediatr Open. 2018;2(1):e000343. doi:10.1136/bmjpo-2018-000343
80. Webbe JWH, Ali S, Sakonidou S, et al. Inconsistent outcome reporting in large neonatal trials: a systematic review. Arch Dis Child Fetal Neonatal Ed. 2020;105(1):69-75. doi:10.1136/archdischild-2019-316823
81. Xue Z, Sun J, Li T, Huang Z, Chen W. How to evaluate the clinical outcome of joint-preserving treatment for osteonecrosis of the femoral head: development of a core outcome set. J Orthop Surg Res. 2019;14(1):317. doi:10.1186/s13018-019-1364-x
