# Supplementary material for: Choosing important health outcomes for comparative effectiveness research: 6th annual update to a systematic review of core outcome sets for research
Source: PLoS One. 2021 Jan 12;16(1):e0244878. doi: 10.1371/journal.pone.0244878 (PMC7802923; doi:10.1371/journal.pone.0244878)
Supplement: S3 Table — (DOCX) [file pone.0244878.s004.docx]

**S3 Table.** COS minimum standards: assessment by study (n=33)

| **Key ✓ = standard met O = unclear whether standard is met X = Standard not met** | | | | | | | | | | | | |
| --- | --- | --- | --- | --- | --- | --- | --- | --- | --- | --- | --- | --- |
|  | **Scope** | | | | **Stakeholders** | | | **Consensus Process** | | | | |
| **Standard number** | **1** | **2** | **3** | **4** | **5** | **6** | **7** | **8** | **9a** | **9b** | **10** | **11** |
| Audigé et al (2019) [1] | ✓ | ✓ | ✓ | ✓ | O | ✓ | X | X | O | ✓ | O | O |
| Blackwood et al (2019) [2] | ✓ | ✓ | ✓ | ✓ | ✓ | ✓ | ✓ | O | O | ✓ | ✓ | O |
| Bogdanet et al (2019) [3] | ✓ | ✓ | ✓ | ✓ | ✓ | ✓ | ✓ | ✓ | ✓ | ✓ | ✓ | ✓ |
| Chen et al (2019) [4] | ✓ | ✓ | ✓ | ✓ | ✓ | ✓ | ✓ | X | ✓ | ✓ | ✓ | ✓ |
| Crudgington et al (2019) [5] | ✓ | ✓ | ✓ | ✓ | ✓ | ✓ | ✓ | ✓ | O | ✓ | ✓ | ✓ |
| de Wolf-Linder et al (2019) [6] | ✓ | ✓ | ✓ | ✓ | ✓ | ✓ | X | X | O | O | O | O |
| Doumouchtsis et al (2019) [7] | ✓ | ✓ | ✓ | ✓ | X | X | X | X | X | X | X | X |
| Gaba et al (2016) [8] | ✓ | ✓ | ✓ | ✓ | ✓ | ✓ | x | O | O | O | O | O |
| Goncalves et al (2019) [9] | ✓ | ✓ | ✓ | ✓ | ✓ | ✓ | ✓ | ✓ | ✓ | ✓ | ✓ | ✓ |
| Haller et al (2019) [10] | ✓ | ✓ | ✓ | ✓ | ✓ | ✓ | x | x | O | O | O | O |
| Harman et al (2019) [11] | ✓ | ✓ | ✓ | ✓ | ✓ | ✓ | ✓ | ✓ | ✓ | ✓ | ✓ | ✓ |
| Healy et al (2019) [12] | ✓ | ✓ | ✓ | ✓ | ✓ | ✓ | ✓ | x | ✓ | O | ✓ | ✓ |
| Hinkelbein et al (2019) [13] | ✓ | ✓ | ✓ | ✓ | ✓ | ✓ | x | x | O | O | O | O |
| Hodgson et al (2019) [14] | ✓ | ✓ | ✓ | ✓ | ✓ | ✓ | ✓ | ✓ | ✓ | ✓ | O | O |
| Ingoe et al (2020) [15] | ✓ | ✓ | ✓ | ✓ | ✓ | ✓ | ✓ | x | ✓ | ✓ | ✓ | ✓ |
| Joachim et al (2020) [16] | ✓ | ✓ | ✓ | ✓ | ✓ | ✓ | ✓ | x | O | O | O | O |
| Krezel et al (2019) [17] | ✓ | ✓ | ✓ | ✓ | ✓ | ✓ | ✓ | x | O | O | O | ✓ |
| Kuizenga-Wessel et al (2017) [18] | ✓ | ✓ | ✓ | ✓ | O | ✓ | ✓ | ✓ | O | O | O | O |
| Lam et al (2019) [19] | ✓ | ✓ | ✓ | ✓ | ✓ | ✓ | ✓ | x | ✓ | ✓ | O | O |
| Mackenzie et al (2020) [20] | ✓ | ✓ | ✓ | ✓ | ✓ | ✓ | ✓ | x | ✓ | ✓ | ✓ | ✓ |
| Perry et al (2019) [21] | ✓ | ✓ | ✓ | ✓ | ✓ | ✓ | ✓ | ✓ | ✓ | ✓ | ✓ | ✓ |
| Pomponio et al (2019) [22] | ✓ | ✓ | ✓ | ✓ | ✓ | ✓ | X | X | O | O | O | O |
| Regardt et al (2019) [23] | ✓ | ✓ | ✓ | ✓ | ✓ | ✓ | ✓ | ✓ | O | O | ✓ | ✓ |
| Rowe et al (2019) [24] | ✓ | ✓ | ✓ | ✓ | ✓ | ✓ | ✓ | ✓ | ✓ | ✓ | O | ✓ |
| Shorter et al (2019) [25] | ✓ | ✓ | ✓ | ✓ | ✓ | ✓ | ✓ | ✓ | ✓ | ✓ | ✓ | ✓ |
| Sun et al (2019) [26] | ✓ | ✓ | ✓ | ✓ | X | ✓ | X | O | O | O | O | O |
| Tong et al (2018) [27] | ✓ | ✓ | ✓ | ✓ | ✓ | ✓ | ✓ | ✓ | ✓ | ✓ | ✓ | ✓ |
| Townsend et al (2019) [28] | ✓ | ✓ | ✓ | ✓ | ✓ | ✓ | ✓ | ✓ | O | O | ✓ | ✓ |
| Van Rijssen et al (2019) [29] | ✓ | ✓ | ✓ | ✓ | x | ✓ | ✓ | x | O | O | O | O |
| van Tol et al (2019) [30] | ✓ | ✓ | ✓ | ✓ | ✓ | ✓ | ✓ | x | ✓ | O | O | ✓ |
| Vicenzino et al (2019) [31] | ✓ | ✓ | ✓ | ✓ | ✓ | ✓ | ✓ | x | ✓ | ✓ | ✓ | O |
| Webbe et al (2019) [32] | ✓ | ✓ | ✓ | ✓ | ✓ | ✓ | ✓ | ✓ | O | O | ✓ | O |
| Xue et al (2019) [33] | ✓ | ✓ | ✓ | ✓ | ✓ | ✓ | ✓ | x | O | O | O | ✓ |

**References**

1. Audigé L, Schwyzer HK, Äärimaa V, Alta TD, Amaral MV, Armstrong A, et al. Core set of unfavorable events of shoulder arthroplasty: an international Delphi consensus process. J Shoulder Elbow Surg. 2019;28(11):2061-71. doi: 10.1016/j.jse.2019.07.021.

2. Blackwood B, Ringrow S, Clarke M, Marshall JC, Connolly B, Rose L, et al. A Core Outcome Set for Critical Care Ventilation Trials. Crit Care Med. 2019;47(10):1324-31. doi: 10.1097/CCM.0000000000003904.

3. Bogdanet D, Reddin C, Macken E, Griffin TP, Fhelelboom N, Biesty L, et al. Follow-up at 1 year and beyond of women with gestational diabetes treated with insulin and/or oral glucose-lowering agents: a core outcome set using a Delphi survey. Diabetologia. 2019;62(11):2007-16. doi: 10.1007/s00125-019-4935-9.

4. Chen K, Andersen T, Carroll L, Connelly L, Côté P, Curatolo M, et al. Recommendations for Core Outcome Domain Set for Whiplash-Associated Disorders (CATWAD). Clin J Pain. 2019;35(9):727-36. doi: 10.1097/AJP.0000000000000735.

5. Crudgington H, Rogers M, Bray L, Carter B, Currier J, Dunkley C, et al. Core Health Outcomes in Childhood Epilepsy (CHOICE): Development of a core outcome set using systematic review methods and a Delphi survey consensus. Epilepsia. 2019;60(5):857-71. doi: 10.1111/epi.14735.

6. de Wolf-Linder S, Dawkins M, Wicks F, Pask S, Eagar K, Evans CJ, et al. Which outcome domains are important in palliative care and when? An international expert consensus workshop, using the nominal group technique. Palliative Med. 2019;33(8):1058-68. doi: 10.1177/0269216319854154.

7. Doumouchtsis SK, Pookarnjanamorakot P, Durnea C, Zini M, Elfituri A, Haddad JM, et al. A systematic review on outcome reporting in randomised controlled trials on surgical interventions for female stress urinary incontinence: a call to develop a core outcome set. BJOG Int J Obstet Gynaecol. 2019;126(12):1417-22. doi: 10.1111/1471-0528.15891.

8. Gaba RC, Lewandowski RJ, Hickey R, Baerlocher MO, Cohen EI, Dariushnia SR, et al. Transcatheter Therapy for Hepatic Malignancy: Standardization of Terminology and Reporting Criteria. Journal of vascular and interventional radiology : JVIR. 2016;27(4):457-73. Epub 2016/02/07. doi: 10.1016/j.jvir.2015.12.752. PubMed PMID: 26851158.

9. Gonçalves AC, Samuel D, Ramsay M, Demain S, Marques A. A Core Outcome Set to Evaluate Physical Activity Interventions for People Living With Dementia. The Gerontologist. 2020;60(4):682-92. Epub 2019/07/28. doi: 10.1093/geront/gnz100. PubMed PMID: 31350898.

10. Haller G, Bampoe S, Cook T, Fleisher LA, Grocott MPW, Neuman M, et al. Systematic review and consensus definitions for the Standardised Endpoints in Perioperative Medicine initiative: clinical indicators. Br J Anaesth. 2019;123(2):228-37. doi: 10.1016/j.bja.2019.04.041.

11. Harman NL, Wilding JPH, Curry D, Harris J, Logue J, Pemberton RJ, et al. Selecting Core Outcomes for Randomised Effectiveness trials In Type 2 diabetes (SCORE-IT): a patient and healthcare professional consensus on a core outcome set for type 2 diabetes. BMJ open diabetes research & care. 2019;7(1):e000700. Epub 2020/01/08. doi: 10.1136/bmjdrc-2019-000700. PubMed PMID: 31908789; PubMed Central PMCID: PMCPMC6936506.

12. Healy P, Gordijn SJ, Ganzevoort W, Beune IM, Baschat A, Khalil A, et al. A Core Outcome Set for the prevention and treatment of fetal GROwth restriction: deVeloping Endpoints: the COSGROVE study. Am J Obstet Gynecol. 2019;221(4):339.e1-.e10. doi: 10.1016/j.ajog.2019.05.039.

13. Hinkelbein J, Iovino I, De Robertis E, Kranke P. Outcomes in video laryngoscopy studies from 2007 to 2017: Systematic review and analysis of primary and secondary endpoints for a core set of outcomes in video laryngoscopy research. BMC Anesthesiol. 2019;19(1). doi: 10.1186/s12871-019-0716-8.

14. Hodgson CL, Burrell AJC, Engeler DM, Pellegrino VA, Brodie D, Fan E, et al. Core Outcome Measures for Research in Critically Ill Patients Receiving Extracorporeal Membrane Oxygenation for Acute Respiratory or Cardiac Failure: An International, Multidisciplinary, Modified Delphi Consensus Study. Crit Care Med. 2019;47(11):1557-63. doi: 10.1097/CCM.0000000000003954.

15. Ingoe HMA, Eardley W, Rangan A, Hewitt C, McDaid C. An international multi-stakeholder delphi consensus exercise to develop a core outcomes set (COS) for surgical fixation of rib fractures. Injury. 2020;51(2):224-9. doi: 10.1016/j.injury.2019.10.031.

16. Joachim KC, Farid-Kapadia M, Butcher NJ, Chee-a-tow A, Monsour A, Cohen E, et al. Core outcome set for children with neurological impairment and tube feeding. Dev Med Child Neurol. 2020;62(2):201-6. doi: 10.1111/dmcn.14326.

17. Krezel AK, Hogg R, Lohfeld L, Chakravarthy U, Azuara-Blanco A. Core outcomes for geographic atrophy trials. Br J Ophthalmol. 2019. doi: 10.1136/bjophthalmol-2019-314949.

18. Kuizenga-Wessel S, Steutel NF, Benninga MA, Devreker T, Scarpato E, Staiano A, et al. Development of a core outcome set for clinical trials in childhood constipation: a study using a Delphi technique. BMJ paediatrics open. 2017;1(1):e000017. Epub 2018/04/11. doi: 10.1136/bmjpo-2017-000017. PubMed PMID: 29637094; PubMed Central PMCID: PMCPMC5842998.

19. Lam TBL, MacLennan S, Willemse PPM, Mason MD, Plass K, Shepherd R, et al. EAU-EANM-ESTRO-ESUR-SIOG Prostate Cancer Guideline Panel Consensus Statements for Deferred Treatment with Curative Intent for Localised Prostate Cancer from an International Collaborative Study (DETECTIVE Study). Eur Urol. 2019;76(6):790-813. doi: 10.1016/j.eururo.2019.09.020.

20. Mackenzie RM, Ells LJ, Simpson SA, Logue J. Core outcome set for behavioural weight management interventions for adults with overweight and obesity: Standardised reporting of lifestyle weight management interventions to aid evaluation (STAR-LITE). Obes Rev. 2020;21(2). doi: 10.1111/obr.12961.

21. Perry H, Duffy JMN, Reed K, Baschat A, Deprest J, Hecher K, et al. Core outcome set for research studies evaluating treatments for twin–twin transfusion syndrome. Ultrasound Obstet Gynecol. 2019;54(2):255-61. doi: 10.1002/uog.20183.

22. Pomponio G, Tedesco S, Peghetti A, Bianchi T, Rowan S, Greco A, et al. Improving the quality of clinical research on chronic wound infection treatment: Expert-based recommendations. J Wound Care. 2019;28:S26-S31. doi: 10.12968/jowc.2019.28.sup1.s26.

23. Regardt M, Mecoli CA, Park JK, de Groot I, Sarver C, Needham M, et al. OMERACT 2018 modified patient-reported outcome domain core set in the life impact area for adult idiopathic inflammatory myopathies. J Rheumatol. 2019;46(10):1351-4. doi: 10.3899/jrheum.181065.

24. Rowe FJ, Hepworth LR, Kirkham JJ. Development of core outcome sets for vision screening and assessment in stroke: A Delphi and consensus study. BMJ Open. 2019;9(9). doi: 10.1136/bmjopen-2019-029578.

25. Shorter GW, Heather N, Bray JW, Berman AH, Giles EL, O’Donnell AJ, et al. Prioritization of outcomes in efficacy and effectiveness of alcohol brief intervention trials: International multi-stakeholder e-delphi consensus study to inform a core outcome set. J Stud Alcohol Drugs. 2019;80(3):299-309. doi: 10.15288/jsad.2019.80.299.

26. Sun HL, Breakey VR, Straatman L, Wu JK, Jackson S. Outcomes indicators and processes in transitional care in adolescents with haemophilia: A Delphi survey of Canadian haemophilia care providers. Haemophilia. 2019;25(2):296-305. doi: 10.1111/hae.13699.

27. Tong A, Manns B, Hemmelgarn B, Wheeler DC, Evangelidis N, Tugwell P, et al. Establishing Core Outcome Domains in Hemodialysis: Report of the Standardized Outcomes in Nephrology-Hemodialysis (SONG-HD) Consensus Workshop. American journal of kidney diseases : the official journal of the National Kidney Foundation. 2017;69(1):97-107. Epub 2016/08/09. doi: 10.1053/j.ajkd.2016.05.022. PubMed PMID: 27497527; PubMed Central PMCID: PMCPMC5369351.

28. Townsend R, Duffy JMN, Sileo F, Perry H, Ganzevoort W, Reed K, et al. Core outcome set for studies investigating management of selective fetal growth restriction in twins. Ultrasound in obstetrics & gynecology : the official journal of the International Society of Ultrasound in Obstetrics and Gynecology. 2020;55(5):652-60. Epub 2019/07/06. doi: 10.1002/uog.20388. PubMed PMID: 31273879.

29. Van Rijssen LB, Gerritsen A, Henselmans I, Sprangers MA, Jacobs M, Bassi C, et al. Core Set of Patient-reported Outcomes in Pancreatic Cancer (COPRAC): An International Delphi Study among Patients and Health Care Providers. Ann Surg. 2019;270(1):158-64. doi: 10.1097/SLA.0000000000002633.

30. van Tol RR, Kimman ML, Melenhorst J, Stassen LPS, Dirksen CD, Breukink SO, et al. European Society of Coloproctology Core Outcome Set for haemorrhoidal disease: an international Delphi study among healthcare professionals. Colorectal Dis. 2019;21(5):570-80. doi: 10.1111/codi.14553.

31. Vicenzino B, De Vos RJ, Alfredson H, Bahr R, Cook JL, Coombes BK, et al. ICON 2019 - International Scientific Tendinopathy Symposium Consensus: There are nine core health-related domains for tendinopathy (CORE DOMAINS): Delphi study of healthcare professionals and patients. Br J Sports Med. 2019. doi: 10.1136/bjsports-2019-100894.

32. Webbe JWH, Duffy JMN, Afonso E, Al-Muzaffar I, Brunton G, Greenough A, et al. Core outcomes in neonatology: Development of a core outcome set for neonatal research. Arch Dis Child Fetal Neonatal Ed. 2019. doi: 10.1136/archdischild-2019-317501.

33. Xue Z, Sun J, Li T, Huang Z, Chen W. How to evaluate the clinical outcome of joint-preserving treatment for osteonecrosis of the femoral head: Development of a core outcome set. J Orthop Surg Res. 2019;14(1). doi: 10.1186/s13018-019-1364-x.
