# Supplementary material for: Choosing important health outcomes for comparative effectiveness research: 6th annual update to a systematic review of core outcome sets for research
Source: PLoS One. 2021 Jan 12;16(1):e0244878. doi: 10.1371/journal.pone.0244878 (PMC7802923; doi:10.1371/journal.pone.0244878)
Supplement: S4 Table — (DOCX) [file pone.0244878.s005.docx]

**S4 Table**. The scope of included studies (n=370)

|  | **Original review**  **n (%)** | **Update review 1**  **n (%)** | **Update review 2 n (%)** | **Update review 3**  **n (%)** | **Update review 4**  **n (%)** | **Update review 5**  **n (%)** | **Update review 6**  **n (%)** | **Combined***    **n (%)** |
| --- | --- | --- | --- | --- | --- | --- | --- | --- |
| **Study aims** |  | | | | | | | |
| Specifically considered outcome selection and measurement | 98 (50) | 21 (75) | 13 (65) | 10 (60) | 33 (69) | 29 (97) | 30 (91) | 234 (63) |
| Considered outcomes while addressing wider clinical trial design issues | 98 (50) | 7 (25) | 7 (35) | 5 (40) | 15 (31) | 1 (3) | 3 (9) | 136 (37) |
| **Intended use of recommendations** | | | | | | | | |
| Clinical research | 176 (90) | 25 (89) | 19 (95) | 11 (73) | 44 (92) | 26 (87) | 24 (73) | 325 (88) |
| Clinical research and practice | 20 (10) | 3 (11) | 1 (5) | 4 (27) | 4 (8) | 4 (13) | 9 (27) | 45 (12) |
| **Population characteristics** | |  |  |  |  |  |  |  |
| Adults | 12 (6) | 12 (43) | 5 (25) | 10 (67) | 21 (44) | 17 (57) | 21 (64) | 103 (28) |
| Children | 22 (11) | 2 (7) | 6 (30) | 0 (0) | 5 (10) | 4 (13) | 4 (12) | 43 (12) |
| Adults and children | 12 (6) | 2 (7) | 0 (0) | 3 (20) | 10 (21) | 2 (7) | 4 (12) | 34 (9) |
| Older adults | 2 (1) | 1 (4) | 0 (0) | 0 (0) | 3 (6) | 2 (7) | 1 (3) | 9 (2) |
| Adolescents and adults | 0 (0) | 0 (0) | 0 (0) | 1 (7) | 4 (8) | 5 (17) | 3 (9) | 13 (4) |
| Not specified | 148 (76) | 11 (39) | 9 (45) | 1 (7) | 5 (10) | 0 (0) | 0 (0) | 168 (45) |
| **Intervention characteristics** | |  |  |  |  |  |  |  |
| All intervention types | 7 (4) | 8 (29) | 12 (60) | 8 (53) | 29 (60) | 17 (57) | 15 (46) | 101 (27) |
| Drug treatments | 39 (20) | 4 (14) | 0 (0) | 0 (0) | 4 (8) | 4 (13) | 1 (3) | 52 (14) |
| Surgery | 13 (7) | 4 (14) | 4 (20) | 4 (27) | 7 (15) | 3 (10) | 4 (12) | 39 (11) |
| Vaccine | 2 (1) | 0 (0) | 0 (0) | 0 (0) | 0 (0) | 0 (0) | 0 (0) | 2 (1) |
| Rehabilitation | 1 (1) | 1 (4) | 0 (0) | 1 (7) | 2 (4) | 0 (0) | 0 (0) | 5 (1) |
| Exercise | 1 (1) | 1 (4) | 1 (5) | 0 (0) | 1 (2) | 0 (0) | 1 (3) | 5 (1) |
| *Exercise (physical activity)* | *1* | *0* | *1* | *0* | *1* | *0* | *1* |  |
| *Exercise (yoga)* | *0* | *1* | *0* | *0* | *0* | *0* | *0* |  |
| Procedure | 4 (2) | 0 (0) | 2 (10) | 0 (0) | 2 (4) | 5 (17) | 3 (9) | 16 (4) |
| Device | 3 (2) | 0 (0) | 0 (0) | 1 (7) | 0 | 0 | 2 (6) | 6 (2) |
| Other | 11 (6) | 5 (18) | 0 (0) | 1 (7) | 3 (6) | 1 (3) | 7 (21) | 28 (8) |
| Not specified | 115 (59) | 5 (18) | 1 (5) | 0 (0) | 0 | 0 | 0 (0) | 116 (31) |

**Additional information provided by updated papers linked to previously published COS are reflected in the combined column*
