# Supplementary material for: Choosing important health outcomes for comparative effectiveness research: 6th annual update to a systematic review of core outcome sets for research
Source: PLoS One. 2021 Jan 12;16(1):e0244878. doi: 10.1371/journal.pone.0244878 (PMC7802923; doi:10.1371/journal.pone.0244878)
Supplement: S5 Table — (DOCX) [file pone.0244878.s006.docx]

**S5 Table.** The methods used to develop COS (n=370)

| **Main methods** | **Original review n (%)** | **Update review 1 n (%)** | **Update review 2 n (%)** | **Update review 3 n (%)** | **Update review 4**  **n (%)** | **Update review 5 n (%)** | **Update review 6 n (%)** | **Combined***  **n (%)** |
| --- | --- | --- | --- | --- | --- | --- | --- | --- |
| **Semi-structured group discussion only** | 55 (28) | 2 (7) | 2 (10) | 0 (0) | 3 (6) | 0 (0) | 0 (0) | 61 (17) |
| **Unstructured group discussion only** | 18 (9) | 0 (0) | 0 (0) | 0 (0) | 0 (0) | 0 (0) | 0 (0) | 18 (5) |
| **Consensus development conference only** | 12 (6) | 0 (0) | 1 (5) | 0 (0) | 1 (2) | 0 (0) | 0 (0) | 14 (4) |
| **Literature/systematic review only** | 11 (6) | 5 (18) | 2 (10) | 1 (7) | 6 (13) | 3 (10) | 1 (3) | 29 (8) |
| **Delphi only** | 6 (3) | 2 (7) | 2 (10) | 0 (0) | 0 (0) | 2 (7) | 0 (0) | 12 (3) |
| **Survey only** | 3 (2) | 0 (0) | 0 (0) | 0 (0) | 1 (2) | 0 (0) | 0 (0) | 4 (1) |
| **NGT only** | 1 (1) | 0 (0) | 0 (0) | 0 (0) | 0 (0) | 0 (0) | 0 (0) | 1 (<1) |
| **Interview only** | 0 (0) | 0 (0) | 0 (0) | 0 (0) | 1 (2) | 0 (0) | 0 (0) | 1 (<1) |
| **Mixed methods *(see descriptions below*)** | 74 (38) | 17 (61) | 13 (65) | 12 (80) | 35 (73) | 25 (83) | 31 (94) | 208 (56) |
| *Delphi + another method(s)* | *22 (11)* | *6 (21)* | *9 (45)* | *9 (60)* | *23 (48)* | *23 (77)* | *29 (88)* | *126 (34)* |
| *Semi-structured group discussion + another method(s)* | *30 (15)* | *7 (25)* | *4 (20)* | *2 (13)* | *9 (19)* | *2 (7)* | *1 (3)* | *53 (14)* |
| *Consensus development conference + another method(s)* | *7 (4)* | *0 (0)* | *0 (0)* | *0 (0)* | *1 (2)* | *0 (0)* | *0 (0)* | *7 (2)* |
| *Literature/systematic review + another method(s)* | *10 (5)* | *4 (14)* | *0 (0)* | *1 (7)* | *2 (4)* | *0 (0)* | *0 (0)* | *17 (5)* |
| *NGT + another method(s)* | *4 (2)* | *0 (0)* | *0 (0)* | *0 (0)* | *0 (0)* | *0 (0)* | *1 (3)* | *4 (1)* |
| *Focus group + another method(s)* | *1 (1)* | *0 (0)* | *0 (0)* | *0 (0)* | *0 (0)* | *0 (0)* | *0 (0)* | *1 (<1)* |
| No methods described | 16 (8) | 2 (7) | 0 (0) | 2 (13) | 1 (2) | 0 (0) | 1 (3) | 22 (6) |

**Additional information provided by updated papers linked to previously published COS are reflected in the combined column*
