# Supplementary material for: Choosing important health outcomes for comparative effectiveness research: 6th annual update to a systematic review of core outcome sets for research
Source: PLoS One. 2021 Jan 12;16(1):e0244878. doi: 10.1371/journal.pone.0244878 (PMC7802923; doi:10.1371/journal.pone.0244878)
Supplement: S6 Table — (DOCX) [file pone.0244878.s007.docx]

**S6 Table.** Participant groups involved in selecting outcomes for inclusion in COS (n=370)

| **Participants category** | **Sub-category (not mutually exclusive)** | **Frequency of participants** |  | |  |  |  | |  |
| --- | --- | --- | --- | --- | --- | --- | --- | --- | --- |
|  |  | **Original review**  **n** | **Update review 1**  **n** | **Update review 2 n** | **Update review 3 n** | **Update review 4 n** | **Update review 5 n** | **Update review 6 n** | **Combined*** |
| **Clinical experts** | | **171/196** | **20/28** | **17/20** | **14/15** | **46/48** | **30/30** | **33/33** | **332/370** |
|  | Clinical experts | 86 | 14 | 16 | 14 | 38 | 27 | 30 | 227 |
|  | Clinical research expertise | 66 | 9 | 9 | 2 | 26 | 14 | 14 | 143 |
|  | Clinical trialists/ Members of a clinical trial network | 9 | 2 |  |  | 1 |  | 2 | 14 |
|  | Others with assumptions* | 54 |  |  |  |  |  |  | 54 |
| **Public representatives** | | **30/196** | **13/28** | **11/20** | **8/15** | **27/48** | **23/30** | **25/33** | **139/370** |
|  | Patients | 18 | 11 | 7 | 8 | 18 | 17 | 21 | 102 |
|  | Carers | 7 | 1 | 3 | 3 | 8 | 8 | 12 | 44 |
|  | Patient support group representatives | 9 | 1 | 4 |  | 9 | 5 | 2 | 33 |
|  | Service users | 2 |  |  | 1 | 2 | 1 | 3 | 9 |
| **Non-clinical research experts** | | **53/196** | **9/28** | **9/20** | **2/15** | **29/48** | **15/30** | **21/33** | **142/370** |
|  | Researchers | 26 | 4 | 4 | 2 | 26 | 12 | 19 | 99 |
|  | Statisticians | 19 | 4 | 3 |  | 1 |  | 3 | 30 |
|  | Epidemiologists | 11 | 2 | 1 |  | 4 | 3 | 1 | 23 |
|  | Academic research representatives | 4 |  |  |  |  | 1 |  | 5 |
|  | Methodologists | 6 | 3 | 2 |  | 4 | 5 | 4 | 26 |
|  | Economists | 3 |  | 1 |  | 2 | 1 | 2 | 9 |
| **Authorities** | | **39/196** | **5/28** | **3/20** | **0/15** | **12/48** | **5/30** | **8/33** | **73/370** |
|  | Regulatory agency representatives | 30 | 4 | 3 |  | 6 | 3 | 2 | 49 |
|  | Governmental agencies | 12 | 1 |  |  | 5 | 1 |  | 19 |
|  | Policy makers | 4 | 1 |  |  | 3 | 3 | 6 | 17 |
|  | Charities | 1 |  |  |  | 1 | 1 | 1 | 4 |
|  | Service commissioners |  |  |  |  | 3 | 1 |  | 4 |
| **Industry representatives** | | **31/196** | **4/28** | **3/20** | **0/15** | **9/48** | **5/30** | **3/33** | **57/370** |
|  | Pharmaceutical industry representatives | 28 | 3 | 3 |  | 8 | 5 | 3 | 52 |
|  | Device manufacturers | 2 | 1 |  |  | 1 | 1 |  | 6 |
|  | Biotechnology company representatives | 1 |  |  |  |  |  |  | 1 |
| **Others** |  | **72/196** | **2/28** | **1/20** | **1/15** | **8/48** | **4/30** | **10/33** | **98/370** |
|  | Service providers |  |  |  |  | 4 |  | 2 | 6 |
|  | Ethicists | 1 |  |  |  |  |  | 1 | 2 |
|  | Journal editors | 2 |  | 1 |  | 2 | 2 | 2 | 9 |
|  | Funding bodies |  | 1 |  |  |  | 2 | 3 | 6 |
|  | Yoga therapists/ instructors |  | 1 |  |  |  |  |  | 1 |
|  | Members of health care transition research consortium |  |  |  | 1 |  |  |  | 1 |
|  | Educationalist |  |  |  |  | 1 |  |  | 1 |
|  | Nutritionist |  |  |  |  | 1 |  |  | 1 |
|  | National professional and academic bodies/ committees |  |  |  |  | 1 |  |  | 1 |
|  | Guideline organisations |  |  |  |  | 1 |  | 2 | 3 |
|  | Social media editors |  |  |  |  |  |  | 1 | 1 |
|  | Social workers |  |  |  |  |  |  | 2 | 2 |
|  | Compensation insurance personnel |  |  |  |  |  |  | 1 | 1 |
|  | NHS manager |  |  |  |  |  |  | 1 | 1 |
|  | Others^^ (besides known participants) | 15 |  |  |  |  |  |  | 15 |
|  | Others with assumptions^ | 54 |  |  |  |  |  |  | 54 |
| **No details given** |  | **24/196** | **7/28** | **3/20** | **0/15** | **0/48** | **0/30** | **0/33** | **33/370** |

**Additional information provided by updated papers linked to previously published COS are reflected in the combined column*

*^ 54 studies with clinical input but unclear about involvement of other stakeholders*

*^^ Workshop/meeting participants (*5), subcommittee/committee (*2), guidelines panel, military personnel, moderator and audience, representatives from EORTC, members with expertise in information technologies, informatics, clinical registries, data-standards development, expertise in vaccine safety, malaria control and representatives from funding agencies/registration authorities, and donor organisation, members of the Rheumatology Section of the American Academy of Pediatrics, the Pediatric Section of the ACR, and the Arthritis Foundation, the diagnostic radiology and basic science communities, and from individuals conversant with functional and quality of life (QOL) assessments, comparative effectiveness research, and cost/ benefit analysis*
