# Supplementary material for: Choosing important health outcomes for comparative effectiveness research: 6th annual update to a systematic review of core outcome sets for research
Source: PLoS One. 2021 Jan 12;16(1):e0244878. doi: 10.1371/journal.pone.0244878 (PMC7802923; doi:10.1371/journal.pone.0244878)
Supplement: S7 Table — (DOCX) [file pone.0244878.s008.docx]

**S7 Table.** Geographical locations of participants included in the development of each COS (n=312)

| **Locations** | **Original review**  **n (%)** | **Update review 1**  **n (%)** | **Update review 2**  **n (%)** | **Update review 3**  **n (%)** | **Update review 4**  **n (%)** | **Update review 5**  **n (%)** | **Update review 6**  **n (%)** | **Combined***  **N (%)** |
| --- | --- | --- | --- | --- | --- | --- | --- | --- |
| North America | 134 (82) | 17 (68) | 9 (64) | 6 (55) | 28 (68) | 19 (73) | 21 (68) | 236 (76) |
| Europe | 125 (76) | 19 (76) | 13 (93) | 10 (91) | 38 (93) | 25 (96) | 28 (90) | 260 (83) |
| Australasia | 42 (26) | 4 (16) | 5 (36) | 3 (27) | 17 (41) | 15 (58) | 17 (55) | 108 (35) |
| Asia | 34 (21) | 3 (12) | 6 (43) | 1 (9) | 18 (44) | 10 (39) | 20 (65) | 96 (31) |
| South America | 16 (10) | 3 (12) | 2 (14) | 1 (9) | 13 (32) | 6 (23) | 12 (39) | 56 (18) |
| Africa | 10 (6) | 1 (4) | 2 (14) | 1 (9) | 7 (17) | 6 (23) | 10 (32) | 39 (13) |
| Total | 164 (84) | 25 (89) | 14 (70) | 11 (73) | 41 (85) | 26 (87) | 31 (94) | 312 (84) |
| No details provided | 32 (16) | 3 (11) | 6 (30) | 4 (27) | 7 (15) | 4 (13) | 2 (6) | 58 (16) |
| Median and range of number of countries | 6, 1-46 | 2, 1-33 | 6, 1-28 | 2, 1-18 | 6, 1-37 | 10, 1-39 | 11, 1-73 | 5, 1-73 |

**Additional information provided by updated papers linked to previously published COS are reflected in the combined column*
