# Supplementary material for: Choosing important health outcomes for comparative effectiveness research: 6th annual update to a systematic review of core outcome sets for research
Source: PLoS One. 2021 Jan 12;16(1):e0244878. doi: 10.1371/journal.pone.0244878 (PMC7802923; doi:10.1371/journal.pone.0244878)
Supplement: S8 Table — (DOCX) [file pone.0244878.s009.docx]

**S8 Table.** Nature of patient participation where detail is reported (n=25)

|  | **Methods used** | **Total number of participants** | **Number of public participants** | **% Public participants when multiple stakeholder groups included** |
| --- | --- | --- | --- | --- |
| 1 | Delphi | R1: 200 | not reported |  |
|  |  | R2: 178 | not reported |  |
|  |  | R3: 161 | 15 | 9 |
|  | Webinar | 31 | 6 | 19 |
| 2 | Delphi | R1: 288 | 41 | 14 |
|  |  | R2: 190 | 19 | 10 |
|  |  | R3:165 | 17 | 10 |
|  | Consensus meeting | 20 | 4 | 20 |
| 3 | Delphi | R1: 93 | 15 | 16 |
|  |  | R2: 97 | 19 | 20 |
|  |  | R3: 107 | 18 | 17 |
|  | Consensus meeting | 19 | 1 | 5 |
| 4 | Delphi | R1: 102 | 26 | 26 |
|  |  | R2: 80 | 19 | 24 |
|  | Consensus meeting | 15 | 6 | 40 |
| 5 | Delphi | R1: 180 | 22 | 12 |
|  |  | R2: 118 | 7 | 6 |
|  |  | R3: 107 | 7 | 6 |
|  | Consensus meeting | 16 | 6 | 38 |
| 6 | Delphi | R1: 37 | 4 | 11 |
|  |  | R2: 37 | 4 | 11 |
|  |  | R3: 69 | 4 | 6 |
| 7 | Delphi | R1: 65 | 7 | 11 |
|  |  | R2: 29 | 4 | 7 |
|  |  | R3: 23 | 2 | 9 |
| 8 | Survey | 18 | 3 | 17 |
|  | Consensus meeting^ | 15 | 0 |  |
|  | Validation survey* | 12 | 12 |  |
| 9 | Delphi* | R1: 301 | 301 |  |
|  |  | R2: 100 | 100 |  |
|  | Focus groups* | 24 | 24 |  |
| 10 | Delphi | R1: 155 | 29 | 19 |
|  |  | R2: 126 | 17 | 14 |
|  | Consensus meeting | 35 | 3 | 9 |
| 11 | Delphi | R1: 38 | 10 | 26 |
|  |  | R2: 33 | 9 | 27 |
|  |  | R3: 33 | 9 | 27 |
| 12 | Delphi | R1: 103 | 28 | 27 |
|  |  | R2: 89 | 18 | 20 |
|  |  | R3: 88 | 18 | 21 |
|  | NGT | 16 | 3 |  |
| 13 | Focus groups* | 36 | 36 |  |
|  | Delphi | R1: 643* | 643 |  |
|  |  | R2: 638 | 510 | 80 |
|  |  | R3: 541 | 410 | 76 |
|  | Meeting | 29 | 2 | 7 |
|  | Consensus meeting | 30 | not reported |  |
| 14 | Delphi | R1: 117 | 17 | 15 |
|  |  | R2: 65 | 7 | 11 |
|  |  | R3: 51 | 5 | 10 |
|  | Consensus meeting | 12 | 2 | 17 |
| 15 | Delphi | R1: 137 | 8 | 6 |
|  |  | R2: 114 | 8 | 7 |
| 16 | Delphi | R1: 501 | 334 | 67 |
|  |  | R2: 422 | 280 | 66 |
| 17 | Delphi* | 18 | 18 |  |
| 18 | Delphi | 60 | 32 | 53 |
|  | Consensus meeting | 17 | 2 | 12 |
| 19 | Delphi | R1: 414 | 244 | 59 |
|  |  | R2: 223 | 84 | 38 |
|  |  | R3: 191 | 61 | 32 |
|  | Consensus meeting | 16 | 5 | 31 |
| 20 | Delphi* | R1: 103 | 103 |  |
|  | Consensus meeting | 30 | not reported |  |
| 21 | Interviews | 29 | 19 | 66 |
|  | Delphi | R1: 91 | 44 | 48 |
|  |  | R2: 95 | 56 | 59 |
| 22 | Delphi | R1: 233 | 153 | 66 |
|  |  | R2: 173 | 116 | 67 |
|  | Consensus meeting | 20 | 13 | 65 |
| 23 | Delphi | R1: 324 | 215 | 66 |
|  |  | R2: 180 | 130 | 72 |
| 24 | Delphi | R1: 103 | 15 | 15 |
|  |  | R2: 92 | not reported |  |
|  |  | R3: 88 | not reported |  |
|  | Consensus meeting | 25 | 6 | 24 |
| 25 | NGT/focus group* | 82 | 82 |  |
|  | *Interviews* | not reported | not reported |  |
|  | Delphi | R1: 1181 | 202 | 17 |
|  |  | R2: 949 | 165 | 17 |
|  |  | R3: 838 | 150 | 18 |
|  | Consensus meeting | 55 | 8 | 15 |

**Public participants only*

**Clinical expert participants only*
