# Supplementary material for: Choosing important health outcomes for comparative effectiveness research: 6th annual update to a systematic review of core outcome sets for research
Source: PLoS One. 2021 Jan 12;16(1):e0244878. doi: 10.1371/journal.pone.0244878 (PMC7802923; doi:10.1371/journal.pone.0244878)
Supplement: S9 Table — (DOCX) [file pone.0244878.s010.docx]

**S9 Table.** Details about COS relevant to 25 leading causes of global disease burden

| **Condition** | **COMET DB search link and date** | **Year of publication** | **Scope** | | | **Stakeholders involved** | | | **Countries involved** |
| --- | --- | --- | --- | --- | --- | --- | --- | --- | --- |
|  |  |  | **Population** | **Interventions** | **Setting** | **Clinical experts** | **Patients/ public** | **Other** |  |
| Neonatal disorders | <http://www.comet-initiative.org/studies/searchresults?guid=b3da3c81-2e69-4c39-a843-ef946199bff4>  (12/11/2020) | 14 relevant COS (8 published and 6 ongoing)  Search terms: Neonatal care | | | | | | | |
| Ischaemic heart disease | <http://www.comet-initiative.org/studies/searchresults?guid=800a4624-4601-46b3-85bc-bff37594d9db>  (12/11/2020) | 2015  (Coronary artery disease) | Age (not specified);  Sex (either) | Any | Clinical trials or clinical research, Clinical practice, Registries | Clinical experts | Patient advocates | Researchers, Policy makers, Service Commissioners | Sweden, UK, USA, Australia, Singapore, India |
|  |  | 2014  (Coronary heart disease) | Age (not specified);  Sex (either) | Procedure (Percutaneous coronary interventions) | Registries | Clinical experts |  | Governmental agencies, Researchers, | Germany, UK, Ireland, Portugal, Spain, Italy, France, Sweden, The Netherlands |
|  |  | 2013  (Coronary heart disease) | Age (not specified);  Sex (either) | Cardioprotective therapies | Clinical trials or clinical research | Clinical experts |  | Other (unknown) | UK, Denmark, Italy, Hungary, Spain, Germany, The Netherlands, France, USA |
|  |  | 2007  (Coronary artery disease) | Age (not specified);  Sex (either) | Device/ Procedure (Drug-eluting coronary stents) | Clinical trials or clinical research | Clinical experts |  | Device manufacturers, Regulatory agency representatives, Researchers | Switzerland, The Netherlands, France, Belgium, Ireland, USA, SIngapore |
|  |  | 2000  (Ischemic heart disease) | Age (not specified);  Sex (either) | Gene/cellular: Coronary angiogenesis | Clinical trials or clinical research | Clinical experts |  |  | Germany, USA |
| Stroke | <http://www.comet-initiative.org/studies/searchresults?guid=62349734-dd09-4b26-946c-f762297fed86> (12/11/2020) | 12 relevant COS (5 published and 7 ongoing)  Search terms: Acute ischemic stroke; Acute stroke; Ischemic stroke; Stroke | | | | | | | |
| Lower respiratory infections | <http://www.comet-initiative.org/studies/searchresults?guid=a7aa3a09-3f5b-476a-9ca7-77bbac342926>  (12/11/2020) | Planning  (Chronic respiratory conditions) | Age (adults);  Sex (either) | Airway clearance techniques | Clinical trials or clinical research, Clinical practice | Clinical experts | Caregivers, Patients, Patient/ support group representatives, Service users | Charities, Researchers, Service providers | Unknown  *Authors (UK)* |
|  |  | Ongoing  (Bronchiolitis) | Age (children, 0-2);  Sex (either) | Any | Clinical trials or clinical research | Clinical experts | Patients, Caregivers | Regulatory agency representatives, Researchers | Unknown  *Authors (Portugal, The Netherlands, Canada)* |
|  |  | Completed – pending publication  (Bronchiolitis) | Age (children, 0-2);  Sex (either) | Other (non-invasive ventilation) | Clinical trials or clinical research, Clinical practice | Clinical experts | Caregivers, Families |  | UK |
|  |  | 2018 (Bronchiectasis) | Age (adults);  Sex (either) | Any | Clinical trials or clinical research | Clinical experts | Patients | Researchers, Funders | 22 countries not reported |
|  |  | 2010  (Influenza) | Age (not specified);  Sex (either) | Complementary and alternative medicine (CAM) therapy | Clinical trials or clinical research | Clinical experts |  | Researchers | USA |
|  |  | 2010  (Hospital-acquired bacterial pneumonia and ventilator-associated bacterial pneumonia) | Age (not specified);  Sex (either) | Drugs | Clinical trials or clinical research | Clinical experts |  | Regulatory agency representatives | USA |
|  |  | 2008 (Community-acquired pneumonia) | Age (not specified);  Sex (either) | Drugs | Clinical trials or clinical research | Clinical experts |  | Academic research representatives, Pharmaceutical industry representatives, Regulatory agency representatives | USA |
|  |  | 2003 (Pneumonia (community-acquired) | Age (not specified);  Sex (either) | Drugs | Clinical trials or clinical research, Clinical practice | Clinical experts |  | Researchers | UK |
|  |  | 1994  (Chronic bronchitis and COPD) | Age (not specified);  Sex (either) | Drugs | Clinical trials or clinical research | Clinical experts |  |  | Spain, Italy, France, UK, Germany, USA |
| Diarrhoeal diseases | <http://www.comet-initiative.org/studies/searchresults?guid=b78a6cbf-c5c2-4480-bb37-e21ee7efac18> (12/11/2020) | 2015  (Acute diarrhoea) | Age (children); Sex (either) | Any | Clinical trials or clinical research | Clinical experts | Caregivers | Researchers, Pharmaceutical industry representatives, Regulatory agency representatives | Hungary, Austria, Romania, Italy, Germany, Sweden, UK, Spain, Latvia, Greece, Croatia, Belgium, Poland, Portugal, Prague, Ukraine, Lithuania, The Netherlands, Turkey, USA, Canada, Costa Rica, United Arab Emirates, Israel, India, Iran, Indonesia, Egypt |
| Chronic obstructive pulmonary disease | <http://www.comet-initiative.org/studies/searchresults?guid=5664fff0-ec14-481b-8579-242d1bd62e66>  (12/11/2020) | Planning (Chronic respiratory conditions) | Age (adults);  Sex (either) | Airway clearance techniques | Clinical trials or clinical research, Clinical practice | Clinical experts | Caregivers, Patients, Patient/ support group representatives, Service users | Charities, Researchers, Service providers | Unknown  *Authors (UK)* |
|  |  | Ongoing | Age (adults);  Sex (either) | Drug | Clinical trials or clinical research | Clinical experts | Patients, patient/support group representatives | Governmental agencies. Methodologists, Pharmaceutical industry representatives, Policy makers, Regulatory agency representatives, Researcher, Service providers | Unknown  *Authors (US)* |
|  |  | Ongoing | Age (adults, 40-99);  Sex (either) | Any | Clinical trials or clinical research, Clinical practice | Clinical experts | Patients, Caregivers, Families, Patient/support group representatives, Service users | Charities, Epidemiologists, Governmental agencies, Methodologists, Pharmaceutical industry representatives, Policy makers, Regulatory agency representatives, Researchers Service providers, Statisticians | Unknown  *Authors (UK, Denmark)* |
|  |  | Ongoing | Age (adults, 40-90);  Sex (either) | Pulmonary rehabilitation | Clinical trials or clinical research, Clinical practice | Clinical experts | Patients, Caregivers, Patient/support group representatives | Researchers, Policy makers, Regulatory agency representatives | Unknown  *Authors (Portugal, Belgium, the Netherlands)* |
|  |  | 2019 | Age (not specified);  Sex (either) | Physiotherapy | Clinical practice | Clinical experts | Patients | Policy makers, Researchers | The Netherlands |
|  |  | 2008 | Age (not specified);  Sex (either) | Pharmacological - drugs | Clinical trials or clinical research | Clinical experts |  |  | UK, Italy, USA, Spain, Netherlands |
|  |  | 1994  (Chronic bronchitis and Chronic obstructive pulmonary disease) | Age (not specified);  Sex (either) | Drugs | Clinical trials or clinical research | Clinical experts |  |  | Spain, Italy, France, UK, Germany, USA |
| Road injuries | <http://www.comet-initiative.org/studies/searchresults?guid=f9917573-5004-4aac-a57a-ed7219e3aaf7> (12/11/2020) | No published/ongoing COS | | | | | | | |
| Diabetes | <http://www.comet-initiative.org/studies/searchresults?guid=05dab5e4-1d61-47b9-9c0b-6b2d4a58d9ec> (12/11/2020) | 16 relevant COS (6 published and 10 ongoing)  Search terms: Diabetes mellitus; Diabetic foot ulcer/ulceration; Diabetic retinopathy (DR); Type 1 Diabetes; Type 2 Diabetes | | | | | | | |
| Low back pain | <http://www.comet-initiative.org/studies/searchresults?guid=492d0048-95ee-4778-a4e8-1bba5739346d>  (12/11/2020) | 1998 | Age (not specified);  Sex (either) | Not specified | Clinical trials or clinical research | Clinical experts |  |  | USA, Canada, Netherlands, UK, Finland |
|  |  | 2003 | Age (not specified);  Sex (either) | Drugs | Clinical trials or clinical research | Clinical experts |  | Pharmaceutical industry representatives, Regulatory agency representatives | Belgium, France, Spain, Austria, Germany,  Portugal |
|  |  | 2015 | Age (adults); Sex (either) | Any | Clinical trials or clinical research | Clinical experts | Patients, Patient/ support group representatives | Researchers | Netherlands, USA, Spain, Australia, Brazil, UK, Norway |
|  |  | Ongoing | Age (adults, 18-99);  Sex (either) | Traditional Chinese medicine | Clinical trials or clinical research, Clinical practice | Clinical experts | Patients, Caregivers, Patient/support group representatives, Service users | Methodologists, Regulatory agency representatives, Researchers, Service providers | China |
|  |  | 2014 | Age (adults);  Sex (either) | Not specified | Clinical trials or clinical research |  |  | Governmental agencies, Other (unknown) | USA |
|  |  | 2015 | Age (adults);  Sex (either) | Any | Clinical practice, Registries | Clinical experts | Patients |  | Unknown |
| Congenital birth defects | <http://www.comet-initiative.org/studies/searchresults?guid=2b7e4377-34aa-4b73-bfce-768b347c63f0> (12/11/2020) | 13 relevant COS (5 published and 8 ongoing)  Search terms: Cerebral palsy; Cleft palate; Congenital abnormalities; Congenital diaphragmatic hernia; Congenital heart disease; Congenital melanocytic naevi; Congenital Pulmonary Airway Malformation (CPAM); Congenital talipes equinovarus (CTEV); Fetal spina bifida; Spina bifida (SB) | | | | | | | |
| HIV/AIDS | <http://www.comet-initiative.org/studies/searchresults?guid=7a324e4f-0811-4946-a2c4-db9be0c0fcc3> (12/11/2020) | Ongoing | Age (adults);  Sex (either) | Any | Clinical practice | Clinical experts | Caregivers, Patients, Patient/ support group representatives | Charities | Unknown |
|  |  | 2001 | Age (not specified);  Sex (either) | Not specified | Clinical trials or clinical research | Clinical experts |  | Researchers | Unknown  *Authors (France, The Netherlands, USA, UK)* |
| Tuberculosis | <http://www.comet-initiative.org/studies/searchresults?guid=574ac13e-8b35-479a-8832-a16a26174389> (12/11/2020) | Ongoing | Age (adults and children);  Sex (either) | Drug | Clinical trials or clinical research | Clinical experts | Patients | Service providers | Unknown  *Authors (China)* |
| Depressive disorders | <http://www.comet-initiative.org/studies/searchresults?guid=dab17638-a584-45cb-9569-3a99b27af40a> (12/11/2020) | Ongoing  (Major depressive disorder) | Age (adolescents, 12-18);  Sex (either) | Any | Clinical trials or clinical research | Clinical experts | Caregivers, Patients | Epidemiologists, Ethicists, Governmental agencies, Journal editors, Methodologists, Researchers, Service providers, Statisticians | Unknown  *Authors (Canada)* |
|  |  | Ongoing  (Major depressive disorder) | Age (adults, 18-65);  Sex (either) | Any | Clinical trials or clinical research, Clinical practice | Clinical experts | Caregivers, Patients, Families, Patient/ support group representatives, Service users | Charities, Economists, Epidemiologists, Methodologists, Researchers, Service providers, Statisticians | Unknown  *Authors (France)* |
|  |  | Ongoing  (Severe mental illness) | Age (adults);  Sex (either) | Self-management | Clinical trials or clinical research, Clinical practice | Clinical experts | Caregivers, Patients, Patient/ support group representatives, Service users | Charities, Economists, Epidemiologists, Methodologists, Researchers, Service commissioners, Service providers, Statisticians | Unknown  *Authors (UK)* |
|  |  | Ongoing  (Common mental health problems) | Age (adults);  Sex (male) | Complex clinical and organisational intervention | Clinical trials or clinical research | Clinical experts | Service users | Researchers | Unknown  *Authors (UK)* |
|  |  | Completed - pending publication | Age (not specified);  Sex (either) | Any | Clinical trials or clinical research | Clinical experts | Caregivers, Patients, Patient/ support group representatives | Governmental agencies, Methodologists, Policy makers, Regulatory agency representatives, Researchers, Service providers | Unknown  *Authors (Sweden)* |
|  |  | Completed - pending publication  (Depression and Anxiety) | Age (children, young people);  Sex (either) | Not specified | Clinical practice | Clinical experts | Patients | Researchers | Australia, Brazil, Chile, Canada, France, Germany, Hong Kong, India, Japan, Nigeria, Singapore, UK, USA |
|  |  | 2017 (Depression and Anxiety) | Age (adolescents, adults);  Sex (either) | Any | Clinical trials or clinical research, Clinical practice | Clinical experts | Patients, Patient/ support group representatives | Methodologists, Researchers | Germany, the Netherlands, Sweden, UK, USA, Canada, Australia, Brazil, Chile, India, Japan, Uganda |
|  |  | 2006  (Major depressive disorder) | Age (not specified);  Sex (either) | Not specified | Clinical trials or clinical research, Clinical practice | Clinical experts |  | Other (unknown) | USA |
| Malaria | <http://www.comet-initiative.org/studies/searchresults?guid=63513db3-bda4-4788-91ad-7106c000283f> (12/11/2020) | 2013 | Age (not specified); Sex (either | Vaccines | Clinical trials or clinical research | Clinical experts |  | Academic research representatives, Epidemiologists, Members of a clinical trial network, Pharmaceutical industry representative, Regulatory agency representative, Statisticians | UK, Switzerland, Belgium, France, Spain, Netherlands, USA, Australia, Gambia, Burkina Faso, Ghana, Mozambique, Mali, Tanzania, Kenya, Malawi, Mali, Senegal |
| Headache disorders | <http://www.comet-initiative.org/studies/searchresults?guid=1285b11d-c4a6-4309-ba19-1d4a3989dfe3>  (12/11/2020) | 2010  (Tension-type headache) | Age (adults); Sex (either) | Drug | Clinical trials or clinical research | Clinical experts |  | Other (unknown) | Denmark, Belgium |
|  |  | 2005  (Headache) | Age (adults, but makes recommendations for children);  Sex (either) | Behavioural therapies | Clinical trials or clinical research | Clinical experts |  | Researchers | USA |
|  |  | 2012  (Migraine) | Age (adults, but provides comments for children);  Sex (either) | Drug | Clinical trials or clinical research | Clinical experts |  | Other (unknown) | Belgium, UK, Denmark, Spain, France, Sweden, Italy, Germany, USA |
|  |  | 2014  (Migraine) | Age (adults);  Sex (either) | Medication - drug | Clinical trials or clinical research |  | Patients |  | The Netherlands |
|  |  | 1995  (Cluster headache) | Age (adults); Sex (either) | Drug | Clinical trials or clinical research | Clinical experts |  | Other (unknown) | USA |
|  |  | Ongoing  (Tension-type headache) | Age (adults); Sex (either) | Traditional Chinese Medicine | Clinical trials or clinical research, Clinical practice | Clinical experts | Patients, Caregivers, Patient/ support group representatives | Journal editors, Researchers, Statisticians | Unknown  *Authors (China)* |
|  |  | Ongoing  (Migraine) | Age (adults); Sex (either) | Complementary and alternative medicine (CAM) therapy | Clinical trials or clinical research, Clinical practice | Clinical experts | Patient/ support group representatives | Epidemiologists, Methodologists, Researchers, Statisticians | Unknown  *Authors (China)* |
|  |  | Ongoing (Headache/ Migraine) | Age (adults); Sex (either) | Any | Clinical trials or clinical research | Clinical experts | Patients, Service users | Charities, Economists, Methodologists, Researchers | Unknown  *Authors (UK)* |
| Cirrhosis | <http://www.comet-initiative.org/studies/searchresults?guid=efdc61f2-c6e3-4dc2-8a47-167ff3bcd584>  (12/11/2020) | No published/ongoing COS | | | | | | | |
| Lung cancer | <http://www.comet-initiative.org/studies/searchresults?guid=2aaa3aee-d6b5-4a47-ac09-c07cfdd7e47c> (12/11/2020) | Ongoing | Age (adults, children); Sex (either) | Complementary and alternative medicine (CAM) therapy, Traditional Chinese Medicine | Clinical trials or clinical research, Clinical practice | Clinical experts | Patients | Conference participants, Researchers | Unknown  *Authors (China)* |
|  |  | Completed | Age (adults, children); Sex (either) | Drug | Clinical trials or clinical research, Clinical practice | Clinical experts | Patients | Conference participant, Epidemiologists, Methodologists, Researchers, Statisticians | Unknown |
|  |  | 2016 | Age (not specified); Sex (either) | Any | Clinical practice | Clinical experts | Patient/ support group representatives | Registry experts | Australia, Belgium, Brazil, The Netherlands, UK, USA |
|  |  | 2015 | Age (adults);  Sex (either) | Not specified | Clinical trials or clinical research, Clinical practice | Clinical experts |  | Epidemiologists, Researchers | Unknown  *Authors (Italy)* |
| Chronic kidney disease | <http://www.comet-initiative.org/studies/searchresults?guid=0c66fecd-3dd9-4ec2-a0db-276e58cfc272>  (12/11/2020) | Ongoing | Age (adults);  Sex (either) | Nutritional and functional foods | Clinical trials or clinical research, Clinical practice | Clinical experts | Patients, Patient/ support group representatives | Conference participants, Ethicists, Food industry, Journal editors, Pharmaceutical industry representatives, Policy makers, Regulatory agency representatives, Researchers, Statisticians | Unknown  *Authors (China)* |
|  |  | Ongoing | Age (adults);  Sex (either) | Any | Clinical trials or clinical research, Clinical practice | Clinical experts | Caregivers, Patients, Families, Patient/ support group representatives | Governmental agencies, Policy makers, Researchers | Unknown  *Authors (Australia, France, Mexico, Cameroon, USA, Hong Kong, Canada, Chile, South Africa, Uruguay, UK)* |
|  |  | 2020 | Age (adults);  Sex (either) | Procedure (Peritoneal Dialysis) | Clinical trials or clinical research | Clinical experts | Patients, Caregivers | Pharmaceutical industry representatives, Policy makers, Researchers | Canada, USA, Hong Kong, Australia, UK, Singapore, Brazil, Belgium, Japan, Bangladesh, Lebanon, Poland, South Korea |
|  |  | Ongoing | Age (children, 0-21);  Sex (either) | Any | Clinical trials or clinical research | Clinical experts | Patients, Caregivers | Pharmaceutical industry representatives, Policy makers, Researchers | Unknown  *Authors (Australia, Canada, USA, the Netherlands, UK, Singapore, India, Belguim)* |
|  |  | Ongoing | Age (adults);  Sex (either) | Procedure (Haemodialysis) | Clinical trials or clinical research, Clinical practice | Clinical experts | Patients, Caregivers, Patient/support group representatives | Charities, Conference participants, Governmental agencies, Guideline developers, Pharmaceutical industry representatives, Policy Makers, Researchers | Unknown  *Authors (Australia, Canada, UK, USA, Belgium, Switzerland)* |
|  |  | 2018 | Age (adults);  Sex (either) | Surgery (Kidney Transplant) | Clinical trials or clinical research | Clinical experts | Patients, Caregivers |  | 73 countries |
|  |  | 2018 | Age (adults);  Sex (either) | Any | Clinical practice | Clinical experts | Patients | Researchers, Epidemiologists, Registry experts, Service providers | 9 countries (not specified) |
| Other musculoskeletal disorders | <http://www.comet-initiative.org/studies/searchresults?guid=c35804b9-1e57-4b27-8b70-05819f310468> (12/11/2020) | 61 relevant COS (49 published and 12 ongoing)  Search terms: Muscle disease; Rheumatology, Musculoskeletal conditions | | | | | | | |
| Age-related and other hearing loss | <http://www.comet-initiative.org/studies/searchresults?guid=02894e2f-e409-48f7-b016-714645ed0544>  (12/11/2020) | Ongoing  (Hearing loss) | Age (children 0-18);  Sex (either) | Any | Clinical trials or clinical research, Clinical practice | Clinical experts | Patients,  Caregivers |  | Unknown  *Authors (UK)* |
|  |  | Ongoing  (Single Sided Deafness, Unilateral Hearing Loss) | Age (adult 16-100);  Sex (either) | Device, procedure | Clinical trials or clinical research, Clinical practice | Clinical experts | Patients,  Patient/ support group representatives,  Service users | Charities, Conference participants, Device manufacturers, Economists, Governmental agencies, Journal editors, Methodologists, Researchers, Service commissioners, Service providers, Statisticians | Unknown  *Authors (UK)*  *Steering group (UK, Belgium, USA)* |
|  |  | Ongoing (Sensorineural hearing loss) | Age (adult, 18-100);  Sex (either) | Any | Clinical trials or clinical research | Clinical experts | Patients, Patient/ support group representatives | Charities, Clinical experts, Device manufacturers, Journal editors, Policy makers, Regulatory agency representatives, Researcher, Service commissioners | Unknown  *Authors (UK)* |
|  |  | Planning (Hearing loss) | Age (not specified);  Sex (either) | Device – cochlear implantation | Clinical trials or clinical research | Unknown | Unknown | Unknown | Unknown  *Authors (UK)* |
| Falls | <http://www.comet-initiative.org/studies/searchresults?guid=b26c7185-5ee7-41ad-9683-a4555b915d7b> (12/11/2020) | 2005 | Age (adults);  Sex (either) | Fall injury prevention | Clinical trials or clinical research | Clinical experts |  | Academic research representatives, Policy makers | UK, Germany, Italy, Denmark, France, Poland, Finland, Sweden, Spain, Norway, Netherlands, USA, Canada, Australia, New Zealand |
| Self-harm | <http://www.comet-initiative.org/studies/searchresults?guid=f42af4f6-8da0-4b73-8c5e-f9ba917c1d31> (12/11/2020) | No published/ongoing COS | | | | | | | |
| Gynaecological diseases | <http://www.comet-initiative.org/studies/searchresults?guid=96f71af6-98d8-4e47-b643-5d62e80b0301>  (12/11/2020) | 24 relevant COS (9 published and 15 ongoing)  Search terms: Gynaecology | | | | | | | |
| Anxiety disorders | <http://www.comet-initiative.org/studies/searchresults?guid=7c651c82-a28e-4cd5-bb2e-6a14422cee34> (12/11/2020) | Ongoing  (Anxiety disorders) | Age (children, 4-18);  Sex (either) | Any | Clinical trials or clinical research | Clinical experts | Caregivers, Patients | Epidemiologists, Ethicists, Governmental agencies, Journal editors, Methodologists, Regulatory agency representatives, Researchers, Service providers, Statisticians | Unknown  *Authors (Canada)* |
|  |  | Ongoing  (Common mental health problems) | Age (adults);  Sex (male) | Complex clinical and organisational intervention | Clinical trials or clinical research | Clinical experts | Service users | Researchers | Unknown  *Authors (UK)* |
|  |  | Completed - pending publication (Depression and Anxiety) | Age (children, young people);  Sex (either) | Not specified | Clinical practice | Clinical experts | Patients | Researchers | Australia, Brazil, Chile, Canada, France, Germany, Hong Kong, India, Japan, Nigeria, Singapore, UK, USA |
|  |  | 2017 (Depression and Anxiety) | Age (adolescents, adults);  Sex (either) | Any | Clinical trials or clinical research, Clinical practice | Clinical experts | Patients, Patient/ support group representatives | Methodologists, Researchers | Germany, the Netherlands, Sweden, UK, USA, Canada, Australia, Brazil, Chile, India, Japan, Uganda |
| Dietary iron deficiency | <http://www.comet-initiative.org/studies/searchresults?guid=31a74ca7-8727-49a3-9f5c-5c408d38692a>  (12/11/20) | Ongoing | Age (adults);  Sex (either) | Preoperative iron supplementation | Clinical trials or clinical research | Clinical experts | Patient/ support group representatives |  | Unknown  *Authors (Germany)* |
|  |  | Ongoing | Age (adults, 16-55);  Sex (female) | Perinatal iron intervention | Clinical trials or clinical research | Clinical experts | Patients | Researchers | Unknown  *Authors (Canada, UK)* |
