# Supplementary material for: Choosing important health outcomes for comparative effectiveness research: 6th annual update to a systematic review of core outcome sets for research
Source: PLoS One. 2021 Jan 12;16(1):e0244878. doi: 10.1371/journal.pone.0244878 (PMC7802923; doi:10.1371/journal.pone.0244878)
Supplement: Attachment — Submitted filename: PLOS ONE review.pdf [file pone.0244878.s011.pdf]

Line	Comment
53	Does “these” refer to the studies, the COS or both?
54	Systematic reviews are usually reviews of studies. It isn’t clear if “a systematic review of COS….” is (a) a systematic review of the studies reporting the development of COS (in which case one might expect judgements to be made about the qualities of those studies, as is usual in any other type of systematic review), or (b) more simply a catalogue of the COSs themselves.<br><br>If it is (a) a better phrase would be “a systematic review of studies reporting the development of COSs”
55 – 62	The content of these lines suggests that it is important to review how COSs have been developed
77	Did they assess “each of the COS…” or did they assess the development of the COS, or more specifically the studies *reporting* the development of the COS? If the latter, there is always the possibility that the quality of the reporting (as distinct from the quality of the development itself) may affect their conclusions.
84	The first aim – to identify new COSs published or indexed in 2019 – is clear and unambiguous.
85	The question: have CYP been included as participants in the development of COSs relevant to CYP? is not an unreasonable one, but does not flow easily from the first. Nor the question about the methods used to facilitate their participation. I think these should be clearly listed as secondary aims of the project.
86	The third aim is also distinct but more related to the first than the one relating to CYP. It is also the first mention of an attempt by the authors to look for “ongoing” COS development exercises.
88	The sentence beginning “Included COS ….” describes a “Method”
92	In any systematic review – even an update – the reader should expect (as a minimum) to know how the included studies were “located, appraised and synthesised”. The inclusion and exclusion criteria should be very clear, and listed bulleted form if necessary
100	The sentence beginning “Studies were eligible for inclusion if…..” is better than the previous sentence which appears redundant. But a clear list would be better in my view.<br><br>I can find no exclusion criteria
135	I am finding it a bit wearrisome continually reading the phrase “as in the previous review update”. My view is that this submitted article should stand alone and the reader should not have to consult the previous papers to know what was done. An “updated” systematic review should be cumulative and provide an up-to-date review of the totality of the literature to date.<br><br>This is quite different from saying – “here are the data from studies that result from searches covering the last 12 months”.
144	The decision to extract information about CYP is interesting in the light of my last comments. The Authors *could* have gone back and extracted these data from all the studies identified in previous iterations of the review. It looks to me as if they have not done that. They are introducing a “change to protocol” here (and at least

	are being explicit about that, so I cannot criticise that) but this really is a separate thread of work compared with the main essence of the project, which is updating their review.
166	I find myself wanting to know which are the 37 new COSs? Looking at ST Table, I cannot work this out.<br><br>The Table focuses (in the first column) on the reports. Why not have the new COS's in column 1 and the reports in a later column?<br><br>Also, why not have a second Table with the studies identified which relate to COSs in the existing database?
